# Integrating genetic variation with deep learning provides context for variants impacting transcription factor binding during embryogenesis

**DOI:** 10.1101/gr.279652.124

**Published:** 2025-05

**Authors:** Olga M. Sigalova, Mattia Forneris, Frosina Stojanovska, Bingqing Zhao, Rebecca R. Viales, Adam Rabinowitz, Fayrouz Hammal, Benoît Ballester, Judith B. Zaugg, Eileen E.M. Furlong

**Affiliations:** 1 European Molecular Biology Laboratory (EMBL), Genome Biology Unit, D-69117 Heidelberg, Germany;; 2 European Molecular Biology Laboratory (EMBL), Structural and Computational Biology Unit, D-69117 Heidelberg, Germany;; 3 Collaboration for Joint PhD Degree between EMBL and Heidelberg University, Faculty of Biosciences, D-69117 Heidelberg, Germany;; 4 Aix Marseille Univ, INSERM, TAGC, 13009 Marseille, France

## Abstract

Understanding how genetic variation impacts transcription factor (TF) binding remains a major challenge, limiting our ability to model disease-associated variants. Here, we used a highly controlled system of F_1_ crosses with extensive genetic diversity to profile allele-specific binding of four TFs at several time points during *Drosophila* embryogenesis. Using a combined haplotype test, we identified 9%–18% of TF-bound regions impacted by genetic variation even for essential regulators. By expanding WASP (a tool for allele-specific read mapping) to examine indels, we increased detection of allelically imbalanced peaks by 30%–50%. This fine-grained “mutagenesis” can reconstruct functionalized binding motifs for all factors. To prioritize causal variants, we trained a convolutional neural network (Basenji) to accurately predict binding from DNA sequence. The model can also predict measured allelic imbalance for strong effect variants, providing a mechanistic interpretation for how the variant impacts binding. This reveals unexpected relationships between TFs, including potential cooperative pairs, and mechanisms of tissue-specific recruitment of the ubiquitous factor CTCF.

The majority of disease-associated genetic variants occur in the noncoding genome (Edwards et al. 2013; Buniello et al. 2019) and likely affect gene regulation (Claringbould and Zaugg 2021). One mechanism by which mutations impact transcriptional programs is through the disruption of transcription factor (TF) binding sites within regulatory elements, such as enhancers and promoters (Wray 2007; Fairfax et al. 2012; Chen et al. 2016; Cannavò et al. 2017; Abramov et al. 2021; Floc'hlay et al. 2021). However, establishing a mechanistic link between DNA sequence variation and TF binding remains challenging (Deplancke et al. 2016). Sequence variation that disrupts the TF's own cognate motif explains only a minority of variable TF binding (Kasowski et al. 2010; Reddy et al. 2012; Ding et al. 2014). For example, only 12% of TF occupancy that varies across genotypes can be explained by variants in that TF's motif in human lymphoblastoid cell lines (Reddy et al. 2012). This implies more complex mechanisms in the majority of cases, including the presence of cryptic low-affinity binding sites (Crocker et al. 2015; Kribelbauer et al. 2019), cooperative binding (Spitz and Furlong 2012; Jolma et al. 2015; Ibarra et al. 2020), and long-range interactions (Rao et al. 2014). In cases in which TFs bind to DNA cooperatively (Spitz and Furlong 2012; Jolma et al. 2015), disruption of one of the two factors’ motif can lead to a loss in binding of the second factor, even though its motif remains intact. At least 7.5% of allele-specific changes in SPI1 (also called PU.1) binding, for example, could be explained by genetic variation in four additional motifs (NFKB1, POU2F2, PRDM1, and STAT2) located in proximity to the SPI1-bound sites (Heinz et al. 2013; Kilpinen et al. 2013; Raza et al. 2014). For some cooperative TF pairs, the binding site is different or a composite of the individual factors motifs (Jolma et al. 2015), or may be driven by DNA shape rather than sequence (Ibarra et al. 2020), and therefore difficult to predict with standard methods, which are typically based on identifying the high-affinity canonical motif (Rube et al. 2022). For other TFs, variants that completely disrupt its motif can have no impact on occupancy, as they remain recruited to the enhancer through protein–protein interactions as a TF collective (Junion et al. 2012; Khoueiry et al. 2017). In addition, the ability of some factors to bind to DNA is altered by changes in chromatin accessibility, for example, through changes in nucleosome occupancy (Barozzi et al. 2014). Thus, cooperative TF interactions, as well as local TF::chromatin interactions, make it difficult to infer the effects of genetic variation based on sequence data alone.

An additional challenge is the confounding effects of both *trans* and *cis* variation (Van De Geijn et al. 2015; Hill et al. 2021). Differences in TF binding across a group of genetically diverse individuals can be caused by the impact of genetic variation on either (or both) the target DNA regulatory sequence (*cis*) or in the TF protein sequence, altering its binding affinity or specificity (*trans* effect). Allele-specific analysis is a very powerful method to tease apart such *cis* and *trans* effects; namely, by considering the ratio between two alleles in heterozygous positions within the same cells, allele-specific measurements will normalize for differences in *trans* factors across individuals. Performing allele-specific analysis in F_1_ hybrids from inbred lines allows for full a priori knowledge of the parental haplotypes and maximizes the variants’ heterozygosity, providing an excellent “mutagenesis” system to link genotype to phenotype in a quantitative controlled manner. Information on allele-specific TF binding can thereby greatly improve functional annotation of noncoding genetic variants associated with disease (Maurano et al. 2012; Cavalli et al. 2016; Walters et al. 2023), as shown in human lymphoblastoid cell lines (Kilpinen et al. 2013; Tehranchi et al. 2016) and patient tissues (Cavalli et al. 2016). In addition to pinpointing direct genetic effects within the TF's motif, F_1_ studies can also give more context by revealing new potential cooperative TF pairs, as shown for three liver-specific factors in mouse F_1_ hybrids (Wong et al. 2017) and three erythroid factors in human cell lines (Behera et al. 2018).

Here, we systematically assessed the effects of *cis* genetic variation (both SNPs and indels) on TF binding during embryonic development, focusing on the recruitment of four essential factors, using *Drosophila* as a model system. By generating eight F_1_ crosses, we analyzed allele-specific binding at multiple embryonic time points (six conditions in total), leading to a total of 60 data sets with chromatin immunoprecipitation followed by sequencing (ChIP-seq), each with biological replicates (120 experiments). To better understand the extent to which genetic variation impacts TF binding of essential regulators in the context of development, we applied a combined haplotype test (CHT) to identify allelically imbalanced peaks. These allelic preferences were sufficient to reconstruct motif binding affinity. To pinpoint the underlying mechanism, we trained a deep neural network, which pinpointed the causal variant for strong effect variants with >90% accuracy. Taken together, this study provides a functional assessment of the sequence requirements for the occupancy of four essential regulators and identifies new dependency relationships for these well-studied factors in the context of embryonic development.

## Results

### Quantifying TF binding across genetically diverse embryos

**Figure 1.**
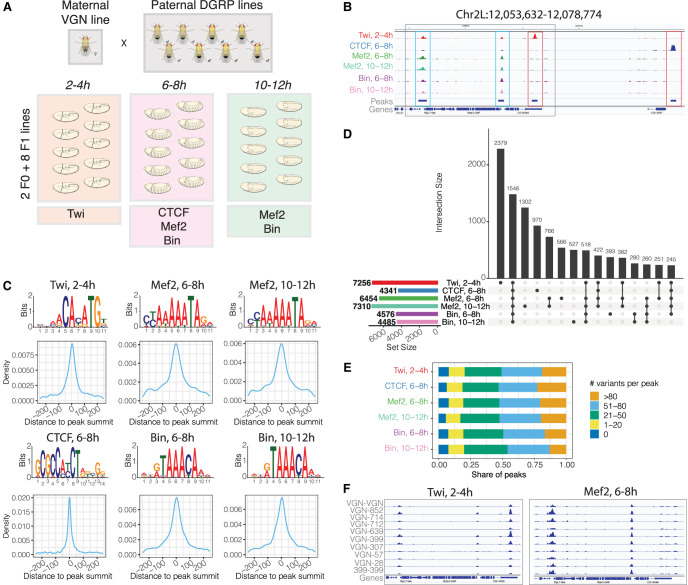
Profiling transcription factor (TF) binding in F_1_ embryos of *Drosophila melanogaster*. (*A*) Experimental design: F_1_ crosses were generated between a maternal VGN line and eight paternal DGRP lines. Staged embryos were collected at three time points (2–4 h, 6–8 h, 10–12 h). Binding of four TFs (Twi, CTCF, Mef2, and Bin) was profiled at the indicated embryonic time points, in two parental lines (VGN and DGRP399) and eight F_1_ lines. (*B*) Occupancy of all TFs in the chromosomal region Chr 2L: 12,053,632–12,078,774. For each TF, tracks were generated by averaging signal across all 10 lines at the corresponding time point. Blue boxes indicate regions cobound by multiple factors; red boxes show peaks specific to one TF. (*C*) De novo motifs discovered in top-1000 ChIP-seq peaks (*top*) and central enrichment of these motifs in full consensus peak sets of Twi at 2–4 h, CTCF at 6–8 h, Mef2 at 6–8 h, Mef2 at 10–12 h, Bin at 6–8 h, and Bin at 10–12 h. (*D*) Overlaps among consensus peak sets for all TFs. (*E*). Consensus peaks overlap for each TF with the indicated number of genetic variants (zero, one to 20, 21 to 50, 51 to 80, more than 80 variants) within 5 kb regions centered on peak summits. (*F*) Binding of Twist at 2–4 h (*left*) and Mef2 at 6–8 h (*right*) across the individual lines on Chr 2L: 12,054,806–12,066,910 (region marked as gray box in panel *B*).

We generated an extensive data set of ChIP-seq for four TFs at several time points during embryonic development in eight F_1_ crosses of inbred lines of *Drosophila melanogaster* and two parental lines (Fig. 1A,B; Supplemental Table S1). All F_1_ crosses share the same maternal line (“virginizer line” [VGN]), providing a controlled genetic background. The eight paternal lines are inbred wild isolates from the *Drosophila* Genetic Reference Panel (Huang et al. 2014) and have extensive genetic variation between them (Fig. 1A; Huang et al. 2014). We also included the maternal VGN line and one of the paternal lines (DGRP-399) in the analysis as controls. The four TFs were selected based on the following criteria. First, three of the TFs (Twist, Mef2, Biniou) are tissue-specific regulators that are essential for mesoderm specification to differentiated muscle and are cobound to many developmental enhancers (Sandmann et al. 2006, 2007; Jakobsen et al. 2007; Zinzen et al. 2009), allowing us to assess the impact of genetic variation on potential cooperative binding. Second, the fourth factor, CTCF, is ubiquitously expressed and has a more general role in 3D chromatin structure and potentially other aspects of gene expression and therefore may have different constraints on its binding (Narendra et al. 2016; Gambetta and Furlong 2018; Dehingia et al. 2022; Li et al. 2022). Third, all four factors have different types of DNA-binding domains—bHLH (Twist), MADS-box (Mef2), forkhead (Biniou), and C2H2 zinc finger (CTCF)—and therefore recognize DNA in different ways, which may influence the impact of genetic variation. Fourth, all four factors are conserved at the protein level from flies to humans (Moon et al. 2005; Potthoff and Olson 2007; Zdobnov et al. 2020), with highly conserved DNA-binding domains (Carlsson and Mahlapuu 2002; Chang et al. 2015).

We genotyped all parental lines to tailor the variant call to our in-house versions of these lines (which necessarily went through further inbreeding and bottlenecks in the years following the original genotyping) to increase sensitivity, by producing 74×–125× genome sequencing coverage across each line (Methods). Variants were called using the GATK4 best-practice pipeline (Poplin et al. 2018); the results were filtered to obtain a stringent variant set used in association tests (to minimize false discovery); and a lenient set was used to correct for mappability issues (to minimize false negatives). A total of 1,735,077 unique heterozygous positions were identified in at least one of the F_1_ individuals (96% biallelic variants) in the stringent variant set (1,912,835 in the lenient), allowing for a genome-wide assessment of the impact of genetic variation on TF binding.

Tightly staged embryo collections, centered on the embryonic stages of key functional importance for each factor, were used for the ChIP-seq experiments (Fig. 1A). For Twist, this was during early embryogenesis (2–4 h, predominantly stage 5–6), in which *twist* is essential for mesoderm gastrulation and its subsequent specification (Supplemental Note 1; Baylies and Bate 1996). *Mef2* and *biniou* have broad expression, spanning many embryonic stages, and are required for the specification and differentiation of somatic (Mef2) and visceral (*Mef2*, *biniou*) muscle. We therefore profiled both TFs at two stages: 6–8 h (predominantly stage 10/11) during cell fate specification and at 10–12 h (predominantly stage 13) at the initiation of differentiation (Fig. 1A,B). This allows for the detection of condition-specific effects of genetic variants for the same factor. The vast majority of sites occupied by CTCF do not change during embryogenesis or even between tissues (Pollex et al. 2024), and we therefore profiled one representative time point at mid-embryogenesis (6–8 h) overlapping Mef2 and Biniou time points.

When mapping ChIP-seq reads from different genetic backgrounds, mapping biases need to be considered. The state-of-the-art tool for handling mapping bias, WASP (Van De Geijn et al. 2015), discards indels. However, because indels span multiple base pairs, they are intuitively more likely to have a stronger impact on TF binding compared with single-nucleotide polymorphisms (SNPs) when overlapping the factor's motif. In addition, masking indels can cause spurious associations in the case of bystander SNPs that are in linkage with causal indels. We identified 305,634 indels in these F_1_ embryos accounting for 17.6% of all of genetic variants, the majority of which are short (69% ≥5 bp, 86% ≥10 bp) (Supplemental Fig. S1A). Because we used paired-end (PE) sequencing for the ChIP-seq, we reasoned that indels could be included without creating mappability issues. Relatively long-read PE sequencing also raises the proportion of reads overlapping indels, thus increasing the number of reads that would be discarded if indels were ignored (Supplemental Fig. S1B,C). To address these issues, we made significant modifications to the original WASP codebase to enable reads to overlap indels. We included further additional options that speed up quantification of genetic variants and increased power to detect errors coming from incorrect genotyping (Methods; http://furlonglab.embl.de/resources/tools). This new version of WASP (WASP-indel) applies the same algorithm to both SNPs and indels, making it equally effective in removing mapping biases caused by either. This increased the usable reads by ∼13%–28% compared with the original software and allowed us to test the effect of 125,432 indels on TF binding, leading to a total of 1,860,509 variants (SNPs and indels) that could be tested (+7.2% of tests). WASP-indel predominantly removed larger indels, indicating that they had the largest impact on mappability (Supplemental Fig. S1D).

After correcting for mapping bias, we performed peak calling applying an irreproducibility discovery rate (IDR; Methods). The majority of samples (96% [116/120]) showed high reproducibility between biological replicates (Methods) (Supplemental Table S2). One replicate of line VGN-DGRP714 failed for both Bin and Mef2 at 10–12 h, and the corresponding samples were excluded. We constructed a consensus ChIP peak set for each condition (TF/time point) using DiffBind (Supplemental Methods) (http://bioconductor.org/packages/release/bioc/vignettes/DiffBind/inst/doc/DiffBind.pdf) by requiring peaks to be present (1 bp overlap) in at least three different lines (1% IDR, conservative set) and recalculated the summits for each peak using counts from all samples. This identified 7256 peaks for Twist 2–4 h, 4341 for CTCF at 6–8 h, 6454 and 7310 peaks for Mef2 at 6–8 h and 10–12 h, and 4576 and 4485 for Biniou at 6–8 h and 10–12 h, respectively (Fig. 1D; Supplemental Table S2). Overall, all lines showed a high correlation between biological replicates over the consensus peak sets, attesting to the quality of the data (Supplemental Fig. S2A). Moreover, de novo motif discovery (Bailey et al. 2009) identified the expected motifs for all factors, which showed strong central enrichment around the consensus peak summits (Methods) (Fig. 1C). The majority of TF peaks in all conditions (87%–96%) (Supplemental Fig. S2B) are in regions of open chromatin, defined by DNase-seq at the same stages of embryogenesis (data from Reddington et al. 2020).

The different TFs had highly overlapping binding (considering peak summits within 500 bp), with 11% (1546/13,693) of the combined consensus peak set being present in all six conditions (Fig. 1D). Four hundred ninety-nine of these cobound regions (32.3%) overlap modENCODE-defined HOT regions (The modENCODE Consortium et al. 2010), whereas the remaining 1047 regions likely reflect true combinatorial binding to regulatory elements, which could be expected for TFs involved in the same tissue's development (Twist, Mef2, Biniou). However, the high overlap of CTCF binding to these putative enhancers was surprising (Fig. 1D). Sites cobound by many factors are enriched at TSS-proximal DHS (within 500 bp from an annotated TSS) and constitutively open distal DHS sites, whereas sites bound by single TFs are more distal and condition -specific, and are likely condition-specific enhancers (Supplemental Fig. S2C,D).

Because of the extensive genetic diversity among these inbred wild isolates, which have a median of one SNP per 227–236 bp, ∼88% of TF peaks have at least one genetic variant within 2.5 kb of the peak summit and 82% have more than 20 variants (Fig. 1E; Supplemental Note 2). As a result, TF binding for each factor is highly variable across genotypes, as shown for two loci for Twist and Mef2 occupancy (Fig. 1F).

### Effects of genetic variation on TF binding are extensive and preferably detected in genomic regions with less buffering

To disentangle the impact of genetic variation on TF binding, we took advantage of the F_1_ experimental design to focus on allele-specific binding, which increases power to detect *cis* effects (McManus et al. 2010; Wong et al. 2017; Link et al. 2018; Floc'hlay et al. 2021). We applied a CHT (Van De Geijn et al. 2015), which simultaneously tests for the effect of each variant on read depth (beta negative-binomial [BNB] component) and allelic imbalance in heterozygous lines (allele-specific [AS] component) using a maximum likelihood approach (Methods). Total and allele-specific read counts were quantified per peak in each of the six consensus peak sets. Using crosses of highly inbred parental lines enables fully resolved haplotype phasing, which allowed us to test variants located ±2.5 kb from the target TF peaks for association, resulting in a total of 4,751,032 autosomal variants, including 45,597 indels. We choose ±2.5 kb to capture not only direct effects (variants within the TFs motif) but also indirect genetic effects such as cooperative interactions (Supplemental Note 3). Importantly, the distribution of allelic ratios (share of reads mapped to one of the two alleles) was highly consistent across samples and centered around 0.5 (Supplemental Fig. S3A), which is the expected allelic ratio under the null hypothesis in CHT (no allelic imbalance), and again supports the quality of the data and the approach.

**Figure 2.**
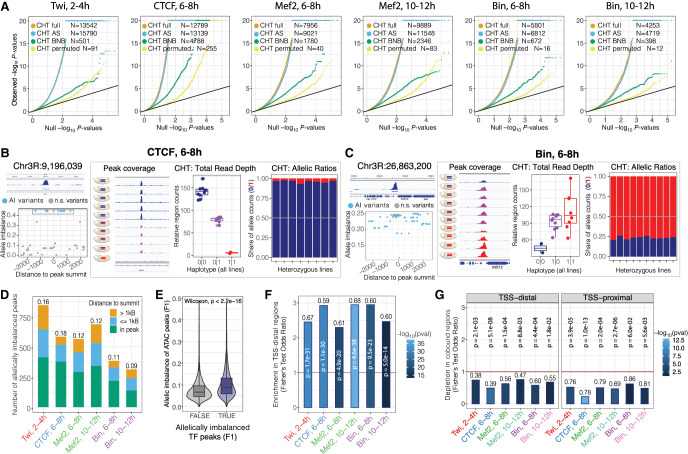
Genetic variation has extensive effects on TF binding during embryogenesis. (*A*) QQ-plots showing CHT results for Twi at 2–4 h, CTCF at 6–8 h, Mef2 at 6–8 h, Mef2 at 10–12 h, Bin at 6–8 h, and Bin at 10–12 h. For each data set, actual *P*-values are plotted against uniform *P*-value distribution for the full CHT (orange), allele-specific (AS) component of the CHT (blue), read depth (BNB) component, and CHT with permuted genotypes (yellow). Number of variants significant at 1% FDR with AI > 0.1 are provided in the *inset*. (*B*) Example of CTCF peak at 6–8 h affected by genetic variation based on CHT. (*Left*, *top*) Browser track for the corresponding peak (average signal across all lines), coordinates of the peak summit is indicated. (*Below*) Allelic imbalance (*y*-axis) for all variants in 2.5 kb radius around peak summit. Significant variants are shown in blue. Top significant variants with the same haplotype (0|1) are highlighted with gray box. For these variants, browser tracks for all lines (*middle*) and two components of CHT are shown (*right*). (*Middle*) Schematic embryos next to browser tracks indicate haplotypes and normalized ChIP signal of the lines. (*Right*) Components of the CHT: normalized total read counts for the corresponding peak in all lines (total read depth; *left*) and share of reads mapped to the reference and alternative alleles in heterozygous lines (allele ratios; *right*). Colors represent genotypes: blue (reference), red (alternative), magenta (heterozygous). (*C*) Same as *A*, for an imbalanced peak of Bin at 6–8 h in the *IntS12* locus. (*D*) Number of allelically imbalanced peaks (*y*-axis) per condition (*x*-axis), defined as peaks with at least one associated significant variant. Fraction of imbalanced peaks in the total number of peaks with genetic variation are shown on *top* of the bars. Colors represent location of the top variant (significant variant with the lowest *P*-value per peak) relative to the peak summit. (*E*) Average allelic imbalance of ATAC-seq peaks overlapping imbalanced and nonimbalanced ChIP-seq peaks from our data set. Allele imbalances of ATAC-seq peaks were quantified in the same F_1_ crosses in the work of Floc'hlay et al. (2021). (*F*) Enrichment of imbalanced peaks in TSS-distal regions (>500 bp from a TSS) per condition (*x*-axis). Fisher's test odds ratios (imbalanced vs. nonimbalanced peaks) are plotted on *y*-axis. Numbers over the bars indicate fraction of TSS-distal imbalanced peaks over all imbalanced peaks. Color represents the Fisher's test *P*-value (−log_10_); the *P*-value is also reported on *top* of each bar. (*G*) Depletion of allele imbalanced peaks in the regions cobound by at least two TFs from our data set for TSS-distal (*left*) and TSS-proximal (*right*) imbalanced peaks. Fisher's test odds ratios (imbalanced vs. nonimbalanced peaks) are plotted on *y*-axis. Numbers over the bars indicate the fraction of cobound imbalanced peaks from all imbalanced peaks. Color represents the Fisher's test *P*-value (−log_10_); the *P*-value is reported over each bar.

We identified strong genetic effects on the occupancy of all factors, with the number of significant variants (FDR < 1%; minimum allelic imbalance = 0.1) ranging between 4253 for Biniou at 10–12 h and 13,542 for Twist at 2–4 h (Fig. 2A). Given the relatively low number of samples in our data set, the allele-specific component (CHT AS, Fig. 2A) had more power than the read depth component (CHT BNB) (Fig. 2A) to detect genetic effects, resulting in a higher number of significant variants. Combining the two components (CHT full) (Fig. 2A) provides a stringent set of significant variants by adding constraints on the total read counts (Supplemental Note 4).

Two examples of allelically imbalanced peaks are shown for CTCF and Biniou binding at 6–8 h (Fig. 2B,C). The panel on the left shows the measurement of allelic imbalance for all significant variants cantered on the ChIP peak at the *pum* and *intS12* loci, respectively. Variants in linkage disequilibrium (LD) have the same allelic imbalance and CHT *P*-values and are highlighted by the gray boxes. Both allelically imbalanced peaks are strongly associated with variants as displayed by the normalized total read counts (Fig. 2B,C, center) and the share of reads mapped to the reference and alternative alleles in heterozygous lines (Fig. 2B,C, right). Despite the small sample size (eight F_1_ crosses), the blocks of LD are relatively contained (e.g., Fig. 2B,C left), with a median of nine significant variants per imbalanced peak.

After testing for association between variants and allelic imbalance, we identified between 9% (Biniou 10–12 h, 313 peaks) and 18% (CTCF 6–8 h, 581 peaks) of TF peaks that are significantly affected by genetic variation (referred to as allelically imbalanced peaks) (Fig. 2D). In 45%–65% of imbalanced peaks, the top significant variant (minimum *P*-value per peak) is inside the peak, whereas the remaining 35%–55% of peaks are affected by more distal genetic variants, within the ±2.5 kb tested window from the TF peak summit (Fig. 2D).

Between 31% and 50% of allelically imbalanced peaks had indels among the significant variants, the majority of which were short (91%–95%<10 bp). Allelic imbalance associated with indels was slightly stronger compared with SNPs in most conditions (Supplemental Fig. S3B), although the difference was unexpectedly low and only significant for Twist at 2–4 h, Mef2 at 6–8 h, and Biniou 10–12 h (Kruskal–Wallis, *P*-value < 0.01).

Our measurements of allelic imbalance in TF binding are further supported by imbalance in the same regions in chromatin accessibility (Fig. 2E; Supplemental Fig. S3C), using ATAC-seq measurements in embryos from the same genetic crosses (Floc'hlay et al. 2021). Moreover, imbalanced TF peaks are strongly enriched in putative enhancers (TSS-distal regions, >500 bp from an annotated TSS; Fisher's test odds ratio 2.2–2.9) (Fig. 2F), extending previous findings for open chromatin and H3K27ac peaks (Floc'hlay et al. 2021). Peaks with allelic imbalance at both putative enhancers (TSS-distal) and promoters (<500 bp from an annotated TSS) are depleted in regions cobound by the other TFs tested here (Fisher's test odds ratios 0.24–0.72) (Fig. 2G) and more generally (using a large collection of ChIP data from the ModERN database) (Supplemental Fig. S3D; Kudron et al. 2018). This suggests that the effect of genetic perturbation is buffered through redundant binding of other factors to the same element, for example, through the collective action of multiple factors to maintain open chromatin. These findings agree with recent observations from single-cell ATAC data in *Mef2* mutant embryos (Secchia et al. 2022) and suggest that the effects of genetic variation are more likely to be detected in genomic regions with less regulatory redundancy. Imbalanced peaks are also enriched in regions with condition-specific (tissue/time point) DHS (Supplemental Fig. S3E). In addition, genes associated with allelically imbalanced peaks (taking the closest gene) are enriched in developmental genes (Supplemental Fig. S3F), consistent with the essential role of these TFs in five out of the six conditions in developmental processes. Imbalanced peaks are also slightly less conserved (Supplemental Fig. S3G).

Overall, our results demonstrate that genetic variation has an extensive impact on TF occupancy during embryogenesis in vivo, even for essential developmental regulators. The effects of genetic variants on TF binding likely depends on the genomic context and complexity of the regulatory region, with promoter regions (TSS-proximal) and cobound regions being less affected (Supplemental Fig. S3D,E).

### Allele-specific binding preferences reconstruct functional TF motifs via in vivo “saturation mutagenesis”

**Figure 3.**
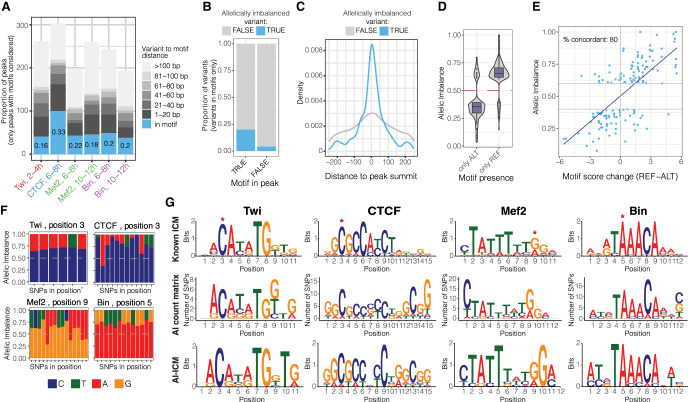
Allele-specific binding provides functionalized TF motif binding preferences at single–base pair resolution. (*A*) Distribution of allele-imbalanced peaks by the shortest distance of significant variants to TF motif (only imbalanced peaks with motifs considered). The fraction of imbalanced peaks with significant variants disrupting TF motif is indicated for each condition (blue color and numbers). (*B*) The fraction of significant (blue) and nonsignificant (gray) variants among all variants in motifs located in and outside TF peaks (*x*-axis). Combined data for all conditions are shown. (*C*) For significant (blue) and nonsignificant (gray) variants in motifs, distribution of variants’ location relative to peak summit. Only motifs in peaks are considered. Combined data for all conditions are shown. (*D*) Allelic imbalance (*y*-axis) of peaks with significant variants in motifs, when the motif is called only in the reference or only in the alternative allele (*x*-axis). Combined data for all conditions are shown; imbalance >0.5 indicates reference allele bias. (*E*) Peak allelic imbalance (*y*-axis) versus motif score change (reference – alternative allele; *x*-axis) for significant variants inside TF motifs. Only cases in which motif is called in both alleles are considered. Combined data for all conditions are shown; variants with an absolute motif score change below one are not shown. The share of concordant variants (variants for which motif score change agrees with direction of allele imbalance) between AI and score change is indicated. (*F*) Allele imbalances associated with variants disrupting TF motifs: Twi at position 3, CTCF at position 3, Mef2 at position 9, and Bin at position 5. Each bar represents a single SNP; colors represent two alleles for each SNP; and the *y*-axis shows allele imbalance. (*G*, *top*) Motif logos of analyzed TFs from known information content matrix (ICM). (*Middle*) Logos represent counts of preferred nucleotides at each position where allelically imbalanced variants occur (allelically imbalanced count matrix). (*Bottom*) Allelically imbalanced count matrices (*middle*) were transformed into information content matrices and visualized as standard motif logos (allelically imbalanced − ICM).

To assess the extent to which allelic imbalance in TF binding is caused by genetic variants (SNPs or indels) within the TFs’ own cognate motif, we scanned positional weighed matrixes (PWMs) of Twist, CTCF, Mef2, and Biniou in 30 bp windows centered on all quantified variants (475,102 autosomal variants) in both the reference and alternative alleles (Methods). This identified a genetic variant in the TF's motif in 16%–33% of imbalanced peaks (Fig. 3A, allelically imbalanced peaks with motifs), which is significantly higher than expected by chance using a matched background of nonimbalanced peaks (10,000 permutation empirical *P*-value < 0.0001 for all conditions) (Supplemental Fig. S4A). Conversely, when variants inside TF motifs were not associated with imbalance, the corresponding motifs were more frequently located either outside the ChIP peak (Fig. 3B) or further away from the peak summit (Fig. 3C), suggesting that they either were false positives of the motif prediction or were not bound by the TF. This highlights how allelic imbalance in a tightly controlled system can pinpoint the likely functional motif within or in the vicinity of a ChIP peak. Overall, all TFs had strong enrichment of allelically imbalanced variants inside their own cognate motif within their ChIP peaks (Fisher's test odds ratios 2.1–3.2) (Supplemental Fig. S4B, left), whereas no enrichment outside the ChIP peaks was observed, except for Twist 2–4 h (Supplemental Fig. S4B, right), attesting to the specificity of the approach. The proportion of allelically imbalanced variants overlapping the cognate motif (16%–33%) (Fig. 3A) is likely an underestimate, as it is based on the identification of the high-affinity canonical site (see Methods). However, as TFs can also bind to low-affinity sites or to alternative motifs (Sandmann et al. 2007; Farley et al. 2016; Kribelbauer et al. 2019), we applied ProBound (Rube et al. 2022), a method to predict TF binding affinity based on equilibrium binding, to the sequences ±30 bp around each variant. This indicates that allelically imbalanced variants are significantly enriched for the presence of binding sites, with a range of affinity scores (Supplemental Fig. S4D). No motifs of other TFs from the Cis-BP database were significantly enriched in imbalanced variants when variants in the TF's own motif were excluded (Supplemental Table S3).

As allelic imbalance is an experimental measure of the quantitative effects of genetic variants on binding, we used this to assess if the differences in binding between alleles are in agreement with the differences in binding affinity as defined by PWM scores. This is akin to a saturation mutagenesis analysis, measuring the impact of all genetic variants within a TF's motif on that factor's binding, across multiple endogenous instances of the motif in an in vivo chromatinized context. Although the base composition of the PWM will not change (as we start from the canonical motif), the relative frequency (or importance) per base could change dramatically. When the motif is completely disrupted to the extent that it is below the detection threshold in one genotype, and thus only present in the alternative genotype, the associated peak has very strong allelic bias (Fig. 3D). When the motif is called in both genotypes, the associated allelic imbalance is correlated with the changes in the motif PWM scores (80% concordant direction) (Fig. 3E). Conversely, using the changes in motif PWM scores (with a score cutoff of one) to predict the direction of allelic imbalance, provides a correct prediction in 68% of cases compared with a random expectation of 50% (Supplemental Fig. S4C). Despite the relatively low number of variants overlapping TF binding motifs (i.e., not reaching saturation), most positions within motifs had SNPs associated with allelic imbalance, thus allowing us to quantify the changes in occupancy (ChIP signal) when each base in the motif is altered. For example, we identified six SNPs disrupting position 3 of the Twist motif at different locations in the genome, and in all cases, allele C was preferred for binding over the alternative A and T alleles (allelic imbalance 0.6–0.7) (Fig. 3F), in agreement with the Twist consensus motif (Fig. 3G, top row). Similarly, in all 13 allelically imbalanced SNPs at position 5 of the Biniou motif, allele A was preferred for occupancy over T and G (allelic imbalance 0.65–0.75) (Fig. 3F). We summarized these results by generating allelic imbalance–based PWMs for all TFs based on the frequencies of their preferred alleles for occupancy at each position (Methods) (Fig. 3G). Reassuringly, these “genetic”-based motif logos are very similar to the affinity-based PWMs for all TFs, which are often generated from in vitro binding assays on naked DNA (Fig. 3G, cf. top and bottom rows). Thus, allele-specific binding information can provide a “functional” assessment of the importance of each base within a TF's motif at single-nucleotide resolution. This will only improve in studies in which more genotypes are assessed.

### Concordant changes in allelic imbalance between TFs and stages indicate a shared regulatory mechanism

Although we found many variants linked to allelic imbalance directly in the TF's motif (discussed above), this can explain only a fraction (16%–33%) of all imbalanced peaks with a recognizable motif, as also observed in previous studies in yeast and humans (Kasowski et al. 2010; Reddy et al. 2012; Ding et al. 2014). This implies widespread indirect effects of genetic variation on TF binding, including the disruption of motifs for a second factor that is cooperatively binding with the first factor. To identify such cases, we searched for allele imbalanced peaks for two or more factors that were coaffected by the same genetic variant, taking advantage of the extensive cobinding among the selected profiled TFs (Fig. 1D).

**Figure 4.**
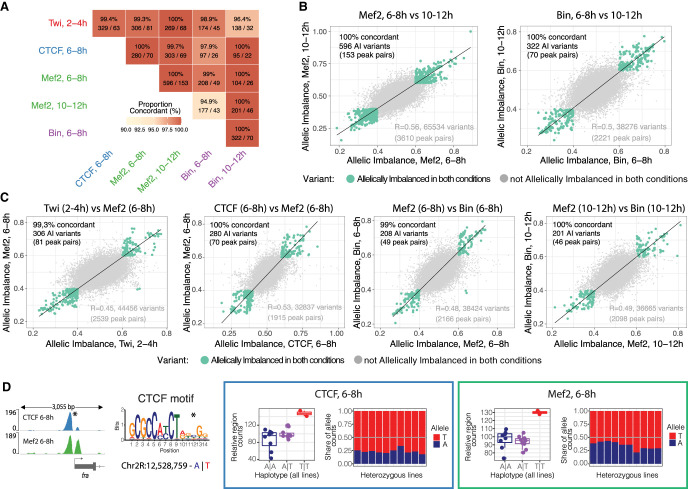
Variants outside the TF's motif might act through disrupted cooperative or collaborative binding. (*A*) Percentage of concordant (same allelic imbalance direction) variants in coaffected peaks for different conditions. Percentage of concordant direction and number of significant shared variants/number of coaffected peaks are indicated. Only variants with unique allelic imbalance values are considered for each pair of coaffected peaks. (*B*,*C*) Correlation in allelic imbalance for variants affecting pairs of conditions: Mef2 at 6–8 versus 10–12 h and Bin at 6–8 versus 10–12 h (*B*); and Twi at 2–4 h versus Mef2 at 6–8 h, CTCF versus Mef2 at 6–8 h, Mef2 versus Bin at 6–8 h, and Mef2 versus Bin at 10–12 h (*C*). Each dot represents a variant (all variants, imbalanced and not imbalanced, with unique allelic imbalance values per peaks overlapping between conditions are considered). Green dots indicate allelically imbalanced variants in both conditions; gray dots, nonimbalanced variants (allelically imbalanced in only one or no conditions). The percentage of concordance in allelic imbalance between conditions (same as in *A*) and the number of variants and coaffected peaks are indicated for allelically imbalanced variants in both conditions (*top left*). Spearman's correlation for all variants (green and gray dots) and total number of variants and total number of peaks overlapping between conditions (*bottom right*). (*D*) Examples of a pair of coaffected peaks for CTCF and Mef2 at 6–8 h, in which one of the TF motifs was disrupted by variants. (*Far left*) Browser tracks showing coaffected peaks (merged across all genotypes per factor) and affected TF motif (affected position marked with an asterisk; reference and alternative alleles are indicated in blue and red, respectively). (*Right*) Boxes represent coverage counts for CTCF (blue) and Mef2 (green) split by allele. In each box, the *left* panel shows the normalized total read counts for the two genotypes for each of the two coaffected peaks; the *right* panel shows allelic ratios (from CHT) in all heterozygous lines. Replicates are included as independent observations.

We identified a number of cases in which the same variant was significantly associated with pairs of allelically imbalanced peaks across time or between factors: 27.6% (153/555) of Mef2 peaks and 23.1% (70/303) of Biniou peaks at two time points, and 18.3% (640/3497) of pairs of different TFs (of 13 TF:time combinations) (Supplemental Fig. S5A). These independent measurements demonstrate the reproducibility of our results and provide further confirmation that these associations are high confidence. They also indicate that many variants impacting Mef2 and Biniou binding do so in a time point–specific manner, leading to significant allelic imbalance only at either 6–8 h or 10–12 h of embryogenesis. This is in keeping with our previous findings of condition-dependent eQTL during embryogenesis, in which the main variants (many in putative enhancers) impacted gene expression only at one embryonic stage (Cannavò et al. 2017). Variants overlapping the coaffected peaks were almost exclusively concordant in the direction of their allelic imbalance (both reference or both alternative bias), whereas there were only few variants that showed opposite imbalanced effects on coaffected peaks (Fig. 4A). When restricting the analysis to the most significant variants for both peaks in the pair, the number of coaffected peaks was lower, but the concordance increased for most pairs (Supplemental Fig. S5B). All coaffected peaks were positively correlated in their allelic imbalance (Fig. 4B,C; Supplemental Fig. S5C–H), which holds true for the same TFs at different time points (r = 0.56 for Mef2 and 0.50 for Biniou), for pairs of mesodermal TFs (r = 0.38–0.49), and even between the insulator protein CTCF and mesodermal factors (r = 0.38–0.53).

In one example, both CTCF and Mef2 binding at 6–8 h are impacted at the promoter region of the *fra* gene (Fig. 4D, left), which shares 30 significant variants with minimum *P*-value for both factors (adjusted *P*-value < 2.2 × 10^−16^). One of those variants (A/T, Chr 2R: 12,528,759) disrupts position 12 of a CTCF motif, resulting in the motif being called only in the alternative allele, even though the affected base in the motif has low information content (Fig. 4D, left). Both TFs have consistent alternative allele bias (Fig. 4D, right), suggesting that the CTCF motif disruption leads to allelic imbalance of CTCF and a muscle-specific TF, Mef2.

However, only a small fraction of coaffected peaks were associated with the disruption of the motif for either of the bound TFs’ (two to 12 peak pairs for different TF combinations) (Supplemental Table S4). This suggests more complex cooperativity or collective binding or, alternatively, low-affinity or completely different motifs for one of the two factors, which are not apparent. Identifying the mechanism is even more challenging as the majority of coaffected peak pairs have multiple associated variants (22 significant variants on average for peak pairs with TF motif disruptions), which further complicates the identification of causal variants even when they affect known TF motifs.

Taken together, our results indicate that the effects of genetic variants on coaffected TF peaks are highly concordant, suggesting the disruption of cooperative or collaborative binding. However, identifying the underlying mechanisms is hampered by the large number of highly significant linked variants in LD and requires further prioritization of significant variants.

### Basenji prioritizes causal variants, contextualizes variant effects, and reveals mechanisms of corecruitment

To help identify the causal variants and elucidate the potential mechanism by which it impacts TF binding, we trained a deep neural network model, Basenji (Kelley et al. 2018), to predict the effect of each variant separately. For training we used an extensive data set of ChIP-seq-bound regions during all stages of the *Drosophila* life cycle, as well as DNase regions during embryogenesis, to learn the sequence rules of bound regions within the wild-type reference *Drosophila* genome. The input training data included 1205 ChIP-seq data sets from ReMap 2022 (Hammal et al. 2022), which we reprocessed to produce RPGC-normalized and background-subtracted bigWig files, when a matching ChIP input was available in the database (Methods) (Supplemental Table S5); 19 DNase hypersensitivity tracks from whole embryos and FACS-purified muscle and neuronal cells (Reddington et al. 2020); and six ChIP-seq tracks from this study obtained by merging F_1_ crosses and replicates within a condition, giving a total of 1230 “occupancy” tracks. The primary objective during training was to predict nonoverlapping binned genomic profiles of the input training data, with a resolution of 128 bp, by utilizing the DNA sequence as the primary source of information. The *Drosophila* dm6 reference genome served as the input for the training process. In essence, the Basenji model seeks to generate accurate and high-resolution genomic tracks by learning the complex relationships between DNA sequences and their corresponding genomic profiles (Kelley et al. 2018). The reference genome was separated into 1041 nonoverlapping (exclusive) sequences with a length of 131 kb. We randomly separated 214 sequences for the validation data set, 193 sequences for the test data set, and the remaining 634 (∼60%) sequences were used for training (Methods).

**Figure 5.**
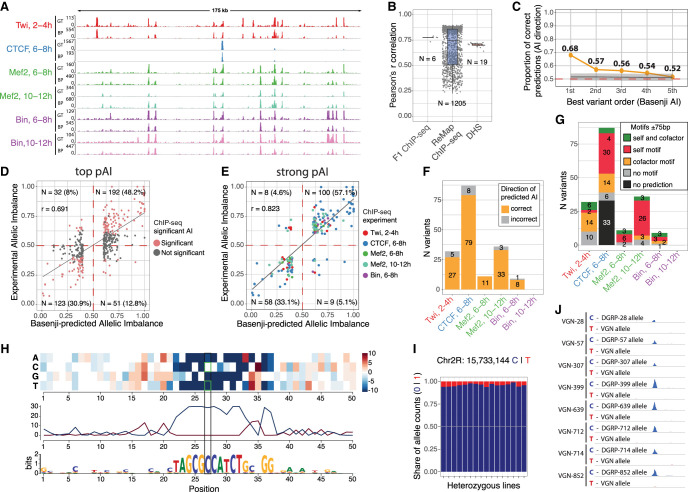
Basenji prioritizes causal variants and predicts disrupted motifs via saturation scores. (*A*) Ground-truth (GT) experimentally measured coverage from merged F_1_ crosses compared with Basenji-predicted (BP) coverage on test sequences from Chromosome 2L. (*B*) Pearson *R* correlation between GT and BP coverage on test set sequences at 1 kb resolution for all sample tracks used in training (ReMap n = 1205, DHS n = 19, F_1_ ChIP-seq n = 6). (*C*) Fraction of correct predictions (same direction of pAI and experimental allelic imbalance [AI]) for variants associated to the same peak and ranked by absolute pAI from Basenji predictions. This test includes all peaks associated with at least one variant with significant experimental allele imbalance. Gray line indicates 10,000 permutations of random variant ranking (background); light gray shadow, 2 SDs. (*D*) Correlation between predicted imbalance and experimentally measured imbalance for variants with the highest absolute pAI per peak (top pAI: absolute pAI > 0.1 for variants ±2.5 kb of each peak); 79.1% of Basenji predictions are in the correct direction with Pearson *R* = 0.691. (*E*) Correlation between strong Basenji pAI (absolute pAI > 0.1) and strong experimental allelic imbalance (absolute AI > 0.1); 90.2% of predictions are in the correct direction. Colors correspond to ChIP-seq samples. (*F*) Counts of strong predictions per ChIP-seq sample divided by correct (same direction of effect; orange) and incorrect (gray) allelic imbalance predictions. (*G*) Same counts as in *F*, with variants colored by motif predictions from saturation mutagenesis ±75 bp around the causal variants: TF's own cognate motif (red), a potential cofactor motif (orange), both the cognate motif and a potential cofactor motif (green), no motif predicted (gray), nonconverging prediction (black). (*H*) Saturation scores around variant Chr 2R: 15,733,144 correlating with high allelic imbalance in CTCF 6–8 h, reveals that Basenji predicts a CTCF motif that is disrupted by a C-to-T variant. (*I*) Proportion of allele-specific reads mapping to reference (C) or alternative (T) alleles on variant Chr 2R: 15,733,144 for CTCF 6–8 h. (*J*) Visualization of ChIP-seq signal separated by maternal and paternal allele on the CTCF peak affected by variant in panel *I*. All paternal lines harbor the C allele, whereas the maternal line has the T allele.

Basenji performs very well in recovering the coverage of all tracks on the validation sequences (Fig. 5A,B), with a median Pearson's correlation of 0.74 for ReMap 2022, 0.70 for DNase, and 0.78 for this study's merged ChIP-seq data (Fig. 5B). Basenji can therefore learn the features of accessible or bound chromatin and is capable of reliably predicting the coverage of TF ChIP-seq tracks in *Drosophila*.

We then used the trained Basenji model to predict the effect of variants on TF binding. Variants were scored for their predicted effect on ChIP-seq signal, allowing us to prioritize variants that caused allelic imbalance. We generated reference and alternative sequences centered around each variant with experimental imbalance. Employing the trained model, we predicted the ChIP-seq peak signal for the reference and alternative sequences and computed a *predicted allelic imbalance* (pAI) score at the variant position. pAI is defined as the predicted coverage on the reference allele divided by the sum of the predicted coverage on reference and alternative alleles. pAI is therefore entirely based on the genomic sequence around the variant and independent of LD.

We assessed whether pAI prioritizes variants linked to the same ChIP-seq peak. For this, we ranked significant variants (from the CHT full test) associated to the same ChIP-seq peak based on their absolute pAI value and then checked the proportion of correct imbalance predictions in each rank (pAI and experimentally measured allelic imbalance >0.5 or <0.5) (Fig. 5C). Looking at each peak's top-ranked variants, the predicted imbalance correctly predicts the direction of experimental imbalance in 68% of cases, indicating that the top predictions are enriched for causal variants (empirical *P*-value after 10,000 variant rankings permutation < 0.0001). This proportion increases to 79.1% for top-predicted variants with large predicted effect size (absolute pAI > 0.1) (Fig. 5D) and decreases to background for lower ranked predicted variants (Fig. 5C). The model's ability to predict the impact of allelic imbalance is greatly improved when the variants overlap the TFs’ own cognate motif. Stratifying variants that overlapped the cognate motif increased the proportion of correct predictions to 91% compared with 64% when no overlap occurred (Supplemental Fig. S6A). The highest-ranked variants per peak (±2.5 kb) are also enriched for overlap with the TFs’ cognate motif compared with lower-rank variants (Supplemental Fig. S6B). In addition, absolute pAI is larger for variants that overlap the cognate motif (median = 0.0174) compared with those that do not (median = 0.0006, Wilcoxon *P*-value < 6.4 × 10^−281^). Taken together, this suggests that pAI can prioritize causal variants among those associated with the strongly imbalanced peaks and that these predictions are partially guided by the presence of TFs cognate motif.

Next, we explored how these predictions can help elucidate biological mechanisms that cause allelic imbalance. We focused on the subset of top-predicted allelically imbalanced variants that also had significant experimental imbalance, referred to as strong predicted allelically imbalanced variants (|AI| > 0.1 and |pAI| > 0.1) (Fig. 5D, red points). First, we observed that strong predicted allelically imbalanced variants inferred the correct direction of effect in >90% of cases (Fig. 5E) with similar precision across conditions (Fig. 5F). In addition, strong predictions of allelic imbalance are enriched (1) at TSS distal peaks (130/175; hypergeometric *P*-value < 4.25 × 10^−21^), (2) at being within a ChIP-seq peak (174/175; hypergeometric *P*-value < 1.72 × 10^−34^), and (3) at overlapping the matching TFs own motif (86/175; hypergeometric *P*-value < 3.83 × 10^−90^) (Supplemental Fig. S6C) compared with all top pAI variants, including those with no experimental imbalance. They are not evenly distributed across TFs, with CTCF representing half of these cases. This may be biological, given the complexity of the CTCF motif (14 bp with high information content) and the strong relationship between its motif presence and binding (Ramírez et al. 2018), which could increase the power of Basenji's predictions for this factor. It may also be technical, as CTCF ChIP-seq tracks are overrepresented in the training set compared with the other three factors, probably allowing Basenji to better model its binding: CTCF tracks represent 2.0% (25 tracks) of all tracks in the training set compared with 0.3% (four) of tracks for Twi and 0.2% (two) of tracks for Mef2 and Bin. The quality of the ChIP data used for training is similarly high for all four factors (median FRiP score of 0.233) (Supplemental Fig. S6D) owing to the sample filters implemented by ReMap (Hammal et al. 2022), and despite the imbalance in the number of ChIP data sets, Basenji performs similarly when predicting the coverage across the six conditions in study (Fig. 5B, F_1_ ChIP-seq; Supplemental Note 5). This suggests that the ability of sequence-based models to predict the impact of genetic variation in TF binding may vary depending on the type of DNA-binding domain of the TF.

To investigate the molecular mechanism of the 175 strong pAIs, we applied in silico saturation mutagenesis (Lee et al. 2015; Kelley et al. 2016) to uncover base pair–specific importance scores and reconstruct relevant motifs around these variants (Fig. 5H). For the majority of strong pAI variants (68% [119/175]), the model predicts at least one motif (either cognate or noncognate) within 75 bp of the strong pAI variants (Fig. 5G). In those cases in which the saturation mutagenesis does not uncover motifs (cognate or potential partner), predictions are more likely incorrect (hypergeometric *P*-value < 0.015) (Supplemental Fig. S6E), indicating that the precision of pAI is dependent on the ability of the model to identify meaningful motifs in the vicinity of the variant. A strong pAI variant overlapping a cognate CTCF motif, for example, has a very strong effect on CTCF binding with an allelic imbalance of 0.925 (Fig. 5H–J) and impacts the central part of the cognate motif, leading to a strong predicted imbalance (0.997) (Fig. 5H). The reference C allele is present across all DGRP lines, whereas the alternative T is only seen in the maternal line, leading to almost complete allelic imbalance at this locus (Fig. 5J).

Among the 175 strong predicted allele imbalanced variants, seven have an effect on two different TFs or stages of embryogenesis (Supplemental Note 6). In all seven cases, the direction of effect is the same between conditions, and pAI could correctly predict the measured imbalance. This suggests cooperative binding. Different TFs were influenced by this to varying extents, again likely reflecting differences in the manner in which they are recruited to DNA. For CTCF and Mef2, the majority of strong pAI variants overlap these factors’ own cognate motifs, rather than another factor's: CTCF pAI variants are linked to 29 CTCF motifs versus nine potential partner motifs; Mef2 pAI to 21 Mef2 motifs versus five partner motifs. This includes four variants associated with changes in Mef2 binding at both 6–8 h and 10–12 h of embryogenesis. This indicates that in the majority of cases, these two TFs do not bind cooperatively but rather depend on the presence of their own motif. However, their occupancy (and motif presence) can influence the occupancy of other factors.

In contrast, Twist appears highly cooperative; only one significant motif variant (one of eight) was in the Twist motif, whereas the others were in potential partner TF motifs within the same ChIP-peaks. This includes significant variants in a Twist imbalanced peak at 2–4 h and also in either CTCF, Mef2, or Biniou imbalanced peaks at 6–8 h. In all cases, the experimental allelic imbalance for Twist and the putative partner's allelic imbalance had concordant directions, suggesting that they bind cooperatively (Supplemental Fig. S7A–C; Supplemental Table S6). Although we lack F_1_ ChIP-seq data for CTCF and Mef2 at the corresponding time point as Twist (2–4 h), we verified that both CTCF (Pollex et al. 2024) and Mef2 (Zinzen et al. 2009) cobind with Twist at 2–4 h at the respective regions, indicating that Twist binding could be facilitated by CTCF and Mef2. In all cases, the TF binding has a stronger allelic imbalance at the later time point, suggesting a downstream effect across embryogenesis (Supplemental Fig. S7A–C). An interesting example is a T-to-C variant with pAI impacting both Twist binding at 2–4 h and Biniou binding at 6–8 h, which does not impact either Twist or Biniou motifs but is positioned in between the two (Supplemental Fig. S7D,E). This may represent an example in which flanking sequences can impact TF binding affinity, for example, by altering DNA shape (Rohs et al. 2009; Yella et al. 2018), and (compatible with this) the sequence between the two motifs might be important for cooperative binding. To explore all saturation mutagenesis results, we created a Shiny app accessible at http://furlonglab.embl.de/data.

### Genetic variation uncovers mechanisms of tissue-specific recruitment of CTCF

Although the majority of genetic variation impacting CTCF is caused by variants within the CTCF motif (29 pAI cases), our results uncovered a number of examples in which CTCF binding is impacted by variants in a lineage-specific TF's motif, including nine predicted potential partner motifs. We uncovered, for example, three cases in which variants in the Mef2 motif are associated with altered Mef2 and CTCF binding at 6–8 h (Supplemental Fig. 8A), as well as five cases in which variants in the Twist motif impact Twist binding at 2–4 h and CTCF binding at 6–8 h (Supplemental Fig. S8B). For CTCF, 6–8 h was the only stage at which we performed the ChIP-seq; however, as the TF is expressed earlier, it is very likely that this SNP would also lead to allelic imbalance in CTCF binding at 2–4 h, or alternatively, Twist binding early in embryogenesis may act to prime this site for CTCF binding at later embryonic stages. CTCF is ubiquitously expressed, whereas Twist and Mef2's expression is tissue specific and restricted to the mesoderm and/or its differentiating muscle. This suggests that tissue-specific TFs may play a role in recruiting CTCF (co-operatively) to a subset of its sites. CTCF binds to the majority of its sites across all tissues (invariant peaks), matching its ubiquitous expression, in both *Drosophila* and mice (Behera et al. 2018; Pollex et al. 2024). However, there is a small fraction of tissue-specific CTCF peaks. Our genetic data measuring allelic imbalance indicate that CTCF is recruited to these sites cooperatively by tissue-specific TFs. Similar findings were also observed across different mouse erythroid cell lines (Behera et al. 2018), suggesting that this is a conserved feature.

## Discussion

In this study, we used F_1_ embryos from crosses of inbred *D. melanogaster* lines to profile allele-specific binding of three essential developmental TFs (Twist, Mef2, Biniou) and one insulator protein (CTCF) at three embryonic time points. This tightly controlled genetic design proved powerful in quantifying allelic imbalance owing to the high homozygosity in the parental lines, resulting in a high density of heterozygosity in the F_1_ crosses, especially considering the small sample size (eight crosses). Our results demonstrate that variants with *cis* effects on TF binding (allele imbalanced peaks) are common, affecting 9%–18% of TF peaks in our data set. This is in the range observed in previous studies in the context of human lymphoblastoid (Kilpinen et al. 2013; Tehranchi et al. 2016) and erythroid (Behera et al. 2018) cell lines or in a tissue (mouse liver) in a differentiated homeostatic state (Wong et al. 2017). Contrary to expectations, we see a similar extent of variation in TF binding for factors that are essential for embryogenesis, in the context of embryogenesis. Because of the large genetic diversity in our F_1_ design, most positions within each TFs’ motif were covered, allowing us to recover the functional importance of each base pair within the motif. Variants in motifs are associated with strong imbalance, and allelic base preferences at these positions are highly concordant with TF binding affinity scores based on PWMs.

Controlling for mapping biases is crucial for measuring allelic imbalance in a reliable way. At the same time, indels prove difficult to assess owing to mappability issues as they create sequence shifts between alleles. We expanded the WASP code to include reads overlapping short indels both at the mappability filters and at allelic imbalance tests. Including indels improved our results by increasing mapped reads by 13%–28%, leading to a substantial increase in the number of imbalanced TF peaks we could detect. As expected, functional variants are enriched for indels (5.8% more likely to be significant compared with SNPs), making them especially relevant to include. On the other hand, the functional impact of indels was similar to SNPs (the change in TF binding was only slightly stronger) (Supplemental Fig. S3B), suggesting that indels with strong phenotypic effects are relatively rare in the genetically controlled context of F_1_ hybrids. This is counterintuitive and suggests that indels with strong effect sizes are most likely selected against in these inbred *Drosophila* lines (Huang et al. 2014), because all lines are homozygous viable, whereas *twist*, *Mef2*, and *biniou* are essential for embryonic development and viability.

### The ability to associate genetic variation to molecular phenotypes (AI) differs depending on the regulatory region and TF involved

Within the genes that have allelic imbalance, different regulatory regions have different sensitivities to the functional impact of genetic variation. Our previous study examining the impact of genetic variation on open chromatin and H3K27ac showed that allelic variation is both more frequent and has greater magnitude at distal regulatory elements (putative enhancers) compared with promoters, despite genetic variation itself being more common at promoters (Floc'hlay et al. 2021). Promoters are also genetically more conserved than distal regions (Cheng et al. 2014).

Even within regulatory elements, different TFs appear to be impacted differently by genetic variation, which likely reflects differences in the way they interact with DNA. For example, the model could learn the rules for sequence changes impacting CTCF binding much better than the other three TFs tested, even though the ChIP data and Basenji's predictions were of similar quality for all four factors. All four TFs have different types of DNA-binding domains, and therefore, different bases are directly contacted by the TF. Although the other three factors have motifs of typical length (6–8 bp), CTCF's motif is much longer and of high information context, and CTCF has a higher retention time on DNA (in the minute range) (Hansen et al. 2017) compared with most other TFs. It may also reflect how cooperative a TF is; we found many examples of variants that impact the binding of multiple factors. This includes variants impacting the motif of tissue-specific factors (Mef2), which also impacted the binding of CTCF, which is a ubiquitously expressed factor. The extent of genetic variation impacting two or more TFs is underestimated here owing to the sample size and is likely much more extensive than currently envisaged. Such differences in the ability to model the impact of genetic variation for different factors will remain a challenge for sequence-based models going forward.

### The mechanism of some genetic variation impacting TF binding remains inaccessible

Although Basenji performed very well, the strongest predictions are biased toward variants in the motif for the bound TF, within the peaks with the strongest measured allelic imbalance. This could be because most causal variants are actually overlapping motifs (cognate or partner), with the model being inherently capable of highlighting them, because the first layer of filters can be traced to motifs. Being completely unbiased toward existing motif knowledge, the model can also recognize unknown motifs and interpret surrounding sequence context, such as DNA shape, and could therefore uncover potential cases of cooperative binding. Although Basenji is capable of generalized variant prioritization, we focused here on a small fraction (5.2% [175/3368]) of highly concordant Basenji predictions to investigate the variants’ mechanism of action. Purely correlative methods (such as the CHT applied here) are much more sensitive in identifying variants associated with allelic imbalance peaks but are likely overestimating the number of causal variants outside peaks and motifs (owing to LD). On the other hand, Basenji is likely underestimating the number of causal variants outside the TFs’ peak that is not linked to LD, as its predictability appears to decrease at increasing distances from the TFs’ motif/peak). Perhaps uncovering such causal variants at greater distances from the TF's peak requires more sophisticated models or the integration of additional types of data such as 3D chromatin proximity (e.g., Hi-C or Capture-C).

In conclusion, pAI offers an orthogonal approach to prioritize causal variants and interpret their effect on TF binding. However, to understand the full spectrum of genetic variation, other methods or models are required to uncover the hidden mechanisms of more distal variation on occupancy and gene expression.

## Methods

### Overview of the data set

Crosses were generated between a single isogenic maternal line (VGN) and eight paternal DGRP lines (DGRP-28, DGRP-307, DGRP-399, DGRP-57, DGRP-639, DGRP-712, DGRP-714, DGRP-852) (MacKay et al. 2012). ChIP-seq was performed on embryos from eight F_1_ and two parental lines (VGN and DGRP-399) as described previously (Bonn et al. 2012) and in the Supplemental Methods, each in two biological replicates, for the following TFs and embryonic time points: Twist at 2–4 h, CTCF at 6–8 h, Mef2 at 6–8 h and 10–12 h, and Biniou at 6–8 h and 10–12 h (six conditions × 10 lines × two replicates = 120 samples in total).

### De novo genotyping of parental lines

*Drosophila* fly lines have a short reproduction cycle, leading to hundreds of generations within a few years. This causes accumulation of de novo variants, creating divergence from original genotyping data, bottleneck effects, and further homozygosity, all of which can also occur in established cell lines. To avoid the need for downstream filtering (gDNA correction) to achieve balanced coverage between parental chromosomes, as used in previous studies (Floc'hlay et al. 2021), we performed deep de novo sequencing of the parental lines and created de novo genotyping of the exact lines used for the experiment to avoid mappability biases from unidentified variants (Supplemental Methods).

Genomic DNA sequencing was performed using an Illumina NextSeq to obtain 150 bp PE reads. The lines were sequenced deeply, giving between 54 million and 92 million reads to achieve a whole-genome coverage that ranged between 74× and 125×. Variant discovery was performed following the GATK best-practice workflow for short variant discovery. Reads were trimmed using Trim Galore! (v. 0.5.0) with options “-q 30 ‐‐phred33 ‐‐fastqc ‐‐illumina ‐‐length 75 –paired.” Reads were then mapped to a joint genome of *D. melanogaster* (dm6) and Wolbachia (AE017196) to quantify the parasite contamination using BWA-MEM (v. 0.7.17) (Li and Durbin 2010) with options “-T 20.” Reads were then sorted using SAMtools (v. 1.9) (Li et al. 2009), and duplicates were first marked with Picard (http://picard.sourceforge.net) MarkDuplicates (v. 2.16.0) and then removed together with reads not mapped in proper pairs using SAMtools with option “-F 1548 -f3.” We then applied GATK (v. 4.1.0.0) (McKenna et al. 2010) base recalibration on the mapped reads, using as a reference the variants included in the full DGRP catalog from 205 inbred fly lines (Huang et al. 2014) with GATK BaseRecalibrator and GATK ApplyBQSR. To increase variant discovery and improve line to line comparisons, we performed a joint variant call using GATK HaplotypeCaller “‐‐min-base-quality-score 20 -G StandardAnnotation.” Variants were then filtered using two sets of cutoffs to produce a list of hard stringent filtered and a more lenient filtered variant sets corresponding to high-confidence and high-sensitivity variant lists, respectively. The hard stringent filtered variant set was obtained using BCFtools (v. 1.9) (Li et al. 2009) with the options “MQ>58 & MQRankSum>−2,5 & MQRankSum<2,5 & QD>20 & SOR<1,5 & FS<10 & ReadPosRankSum>−4 & ReadPosRankSum<4”, whereas the lenient filtered variant set was obtained using the options “MQ>40 & MQRankSum>−12,5 & MQRankSum<12,5 & QD>2 & SOR<3 & FS<60 & ReadPosRankSum>−8 & ReadPosRankSum<8”.

### ChIP-seq mappability and inclusion of indels: WASP-indel

FASTQ files with ChIP-seq reads were preprocessed as described in the Supplemental Methods. Because the ChIP-seq samples came from genetically diverse lines of *D. melanogaster*, mapping reads to the reference genome could result in some reads being mapped better to the reference allele (reference bias). To correct for such potential mapping biases, we adapted the original WASP code from Van De Geijn et al. (2015) to include indels into the mappability correction (the original version discarded reads with indels). WASP-indel is available at http://furlonglab.embl.de/resources/tools.

In brief, all mapped reads that overlapped at least a SNP or indel in the parental *Drosophila* lines (from the de novo genotyping, lenient variant set) were identified. Alleles in those reads were substituted with all possible allele combinations from the parental VCF file (up to 64 different allelic combinations per read, default) and mapped back to the reference genome with the same options as specified above. If all combinations of the reads overlapping variants mapped with a mapping quality greater than 10 uniquely to the same position of the reference genome as the original read, they were retained for subsequent analysis. Finally, duplicate reads were removed with the Je suite (Girardot et al. 2016), using random filtering of duplicates to avoid reference bias as previously discussed (Van De Geijn et al. 2015).

### Phased genotypes for F_1_s

Phased genotypes for the stringent F_1_ variant set were constructed with a custom script (construct_phased_vcf_with_replicates_with_gl_tabix.py) using genotypes of parental lines. Only variants without missing information that were homozygous in all nine parental lines were considered (1,799,462 variants considered, 96% of all 1,878,415 variants). In addition, we kept only biallelic variants for subsequent analysis (1,735,077 variants, 96% of the above).

### Total and allele-specific counts per ChIP peak

ChIP peaks were called using MACS2 (Zhang et al. 2008), implementing an IDR pipeline (Li et al. 2011), as described in the Supplemental Methods. Allelic counts for each variant in the final VCF file were calculated using get_counts.py script from WASP-indel. We required a minimum of 400 reads mapped in the target region (total across all lines) and a minimum of 50 allele-specific reads. Variants in 2.5 kb radius of consensus peak summits (target regions) were therefore defined as test variants (get_target_regions.py script with parameters: min_read_count = 400, min_as_count = 50, min_het_count = 1, min_minor_allele_count = 1). For each test variant and target region, total and allele-specific read counts were calculated with get_region_data.py script. Script update_total_depth.py from the original WASP pipeline (Van De Geijn et al. 2015) and used to adjust total counts for the GC content and the fraction of reads in peaks.

### Combined haplotype test

The CHT from the WASP suite (Van De Geijn et al. 2015) was used to define variants associated with differences in read depth or/and allelic imbalance among individuals. Allele-specific, beta-binomial (AS) and read depth, beta-negative binomial (BNB) are the two components of the CHT: The former models the allelic imbalance at phased heterozygous variants, whereas the later models the total read depth in the target region. These two components are linked by the shared parameters that define the effect sizes of the test variants, and combining them in the CHT provides more confidence in the identification of genetic variants with significant effects on TF binding. CHT was run separately for each of the six biological conditions (Twist at 2–4 h; Mef2, CTCF, and Biniou at 6–8 h; Mef2 and Biniou at 10–12 h). To increase the power of the test, we treated biological replicates as individuals and included both parents (only containing homozygous positions) and F_1_s (both homo- and heterozygous positions). At 10–12 h, line VGN-DGRP714 was removed (see above), resulting in the set of 18 samples (20 samples at 2–4 h and 6–8 h). Only variants on autosomes were considered for the CHT. We ran CHT, allele-specific (beta-binomial), and read depth (BNB) parts of the CHT on real data and permuted genotypes. *P*-values from all these tests were plotted against expected *P*-value distributions (Fig. 2A). The full CHT results are available at http://furlonglab.embl.de/data.

### Quantifying variant effects

Adjusted *P*-values (FDR, BH correction) were calculated on the sets of variants quantified by CHT; an FDR threshold of 0.01 was used to define significant variants. Reference allele bias was defined for each variant as $\frac{\alpha }{\alpha +\beta }$, where *α* and *β* are expected counts for reference and alternative alleles (from CHT), respectively. Allelic imbalance (AI) was defined for each variant as $\;\mathrm{AI}=\;\mathrm{abs}\left(0.5-\;\frac{\alpha }{\alpha +\beta }\right)$, where *α* and *β* are defined above, and 0.5 is the expected allelic ratio in case of no imbalance. A threshold of AI = 0.1 was used to define strongly imbalanced significant variants. Relative region read counts were defined as 100 × (total region read counts) / (expected region read counts), where expected region read counts were estimated with update_total_depth.py script from the WASP suite.

Gene Ontology enrichment and motif analysis of allelic imbalance peaks were performed using standard procedures, as described in the Supplemental Methods.

### Basenji model and training

The input of the model is nonoverlapping sequences from the reference dm6 genome. We extracted 1041 nonoverlapping sequences with a length of 131 kb across the genome, of which 214 sequences were randomly separated for the validation set, 193 sequences for the test set, and the remaining 634 sequences for a train set. With the fixed input size of 131,072 bp, for better learning, 8192 bp were cut out from both ends of the input, and the training loss was calculated only on the center crop. The center crop then has a length of 114,688 bp. The output of the model comprises 1230 tracks, of which 1205 are derived from the ReMap tracks (see Supplemental Methods, “ReMap 2022 coverage tracks” section); 19 are DHS tracks; and the remaining six are from the F_1_ ChIP-seq tracks generated in this study.

During the training phase, we used random shift and reverse complement as data augmentation techniques. For the random shift, the fixed input size of 131,072 bp was reduced to 114,688 bp, and the center of this crop was subjected to the random shift. The reverse complement randomly selected half of the training sequences in the mini-batch and replaced them with their reverse complement sequence.

The first part of the model is a convolutional block followed by a max-pooling operation with a pool size of two. The convolutional blocks consist of the GELU activation function, convolution with a kernel of width 15 and 288 filters, and batch normalization. These convolution and pooling operations aggregate the base pair information into 128 bp bins. The convolutional block is repeated six times with a kernel of width five and increasing the number of filters from an initial 288 by 1.1776× each block to 768 filters by the end. Subsequently, 11 layers of residual blocks with dilated convolution are applied, followed by a final convolutional layer to make the prediction. The residual block comprises a GELU activation, a dilated convolutional layer with a kernel of width three and 384 filters, batch normalization, another GELU activation, a convolutional layer with a kernel of width one and the original number of filters, batch normalization, and dropout with a 0.3 rate. Each residual block features a skipped connection to the inputs of the block. The final output convolutional layer undergoes average pooling, and a dense layer of size 1230 with softplus activation produces the model predictions.

The training of the model was performed with stochastic gradient descent (SGD) with a learning rate of 0.1. The batch size was set to four, and the momentum was configured at 0.99. Additionally, a clip norm of two was employed to control the gradient norm, preventing it from growing too large during training. The model training was executed on NVIDIA A100 graphics processing unit (GPU).

### pAI and variants prioritization

For each variant, we created two sequences, a reference and alternative allele, centered at the position of the variant. The reference allele contains the information from the dm6 genome, and an alternative sequence introduces the variant in the sequence. The trained Basenji model was used to create predictions for the reference allele and the alternative allele individually. The pAI corresponds to the Basenji prediction for the reference allele divided by the sum of the Basenji prediction for the reference and the alternative alleles. If multiple variants had a significant association to the same peak (AI > 0.1 and FDR < 0.01), they were prioritized by ranking pAI (|pAI − 0.5|).

### Saturation mutagenesis

We performed saturation mutagenesis applying a “basenji_sat_bed.py” script within Basenji's repository (Kelley et al. 2018). The saturation mutagenesis was calculated for the six condition merged tracks (merge of crosses and replicates) that provide a baseline coverage. We used “sum” as the prediction statistics and performed saturation mutagenesis 75 bp upstream of and downstream from the 176 variants that have both AI and pAI > 0.1. The saturation scores were then normalized on the average of all score absolute values, and the deltas were computed by subtracting the scores for the reference allele at each base pair. The importance scores represent the sum of the absolute values of deltas per base pair. We created the motif logos from the importance scores using R (R Core Team 2021) package motifStack (Ou et al. 2018).

## Data access

All raw data, which consist of more than 120 demultiplexed files, were submitted to EMBL-EBI ArrayExpress (https://www.ebi.ac.uk/biostudies/arrayexpress) under accession numbers: E-MTAB-14209 (F_1_ ChIP-seq) and MTAB-14210 (DNA sequencing data for variant calling). The processed F_1_ bigWigs, reprocessed ReMap bigWigs, the de novo variant calls, the Basenji model, and other data are available on the Furlong laboratory web page at http://furlonglab.embl.de/data. All the code referenced in the Methods section is available at the EMBL Git server https://git.embl.de/forneris/f1-chip-seq-drosophila and as Supplemental Code.

## Supplemental Material

Supplement 1

Supplement 2

Supplement 3

Supplement 4

Supplement 5

Supplement 6

Supplement 7

Supplement 8
