# Supplementary material for: Integrating genetic variation with deep learning provides context for variants impacting transcription factor binding during embryogenesis
Source: Genome Res. 2025 May;35(5):1138–53. doi: 10.1101/gr.279652.124 (PMC12047541; doi:10.1101/gr.279652.124)
Supplement: Supplement 7 [file Supplemental_Material.pdf]

Over view of Supplemental Material

Supplemental Figures

Supplemental Fig. 1: ChIP-seq data QC and mappability
Supplemental Fig. 2: Properties of transcription factor ChIP peaks
Supplemental Fig. 3: Properties of Allelic imbalance in transcription factor peaks
Supplemental Fig. 4: Overlap of AI associated significant variants in the TFs' cognate motif
Supplemental Fig. 5: Concordant changes in AI for variants affecting two TFs
Supplemental Fig. 6: Basenji predictions of the impact of genetic variation on TF binding
Supplemental Fig.7: Basenji saturation mutagenesis around variants associated to multiple peaks
Supplemental Fig. 8: Examples of allelic imbalance in a TF motif impacting two TF binding events

Supplemental Data Tables

Information describing the contents of Supplemental Data Tables S1-S6 is provided with the tables

Supplemental Notes

Note 1: Additional informaiton on the function of the TF *twist*
Note 2: Genetic diversity and LD in *Drosophila* lines
Note 3: The selection of ±2.5kb windows
Note 4: Relatedness between lines does not lead to an excess of false associations
Note 5: Imbalanced training sets for deep learning
Note 6: Variants impacting allelic imbalance in two conditions (factors or time)

Supplemental Methods

Drosophila genetics and embryo collection
ChIP-seq on *Drosophila* embryos
Genomic DNA sequencing of parental lines
ChIP-seq read mapping and peak calling
ChIP Peak calling and Irreproducibility Discovery Rate (IDR) analysis
Allelic imbalanced peak Gene Ontology enrichment and conservation
Motif analysis
ReMap 2022 coverage tracks

Bibliography

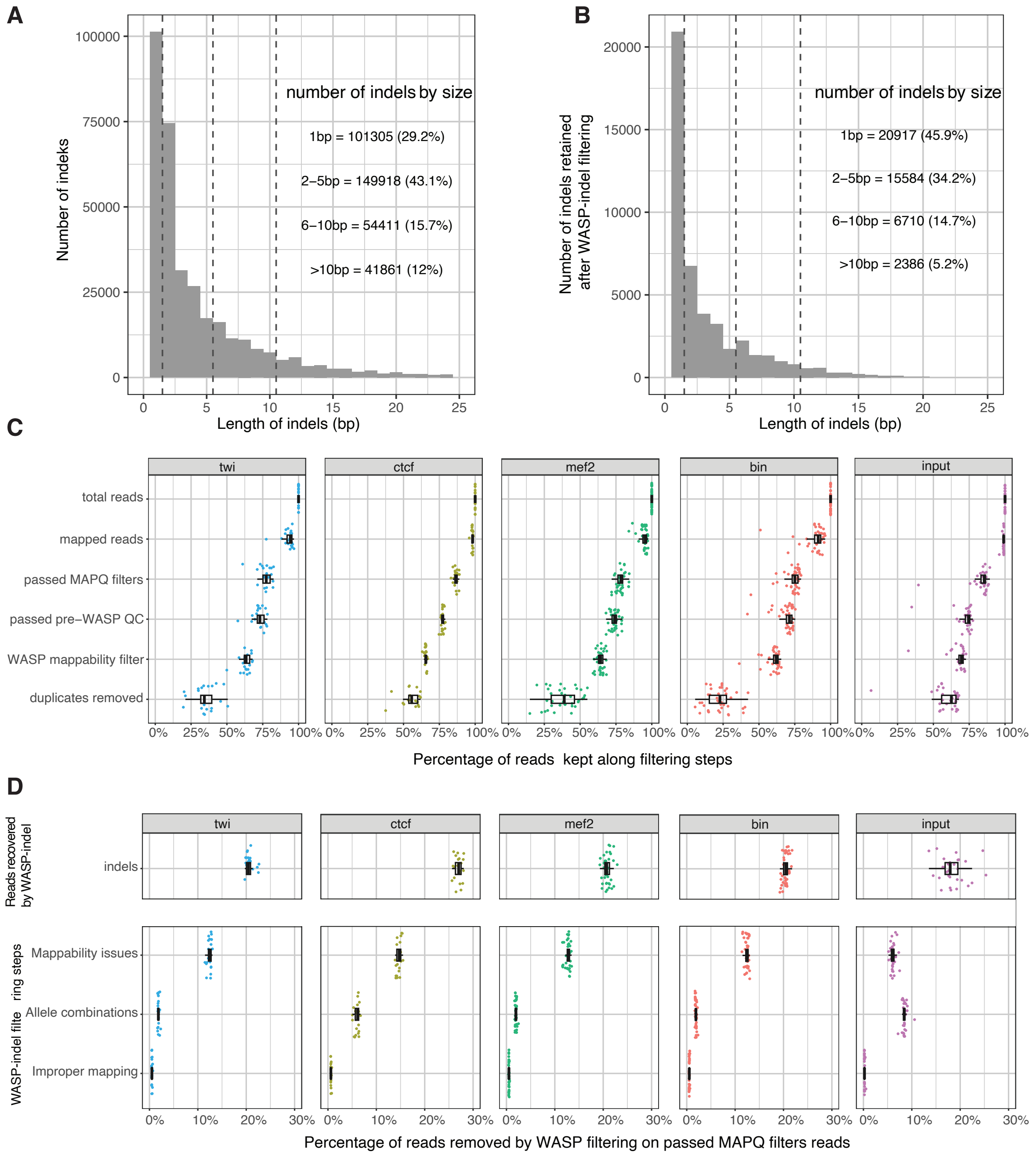

Supplemental Fig. 1: ChIP-seq data QC and mappability

A. Distribution of indels length in the stringent variant call. **B.** Distribution of indels length within ±2.5kb of a ChIP-seq peak after WASP-indel filtering. **C.** Percentage of reads remaining after QC filtering steps in the ChIP-seq mapping pipeline (see Methods for steps). *Total reads*: Proportion of reads in the raw fastq files before mapping and filtering (100% by definition). *Mapped reads*: proportion of reads properly mapped to the reference genome. *Passed MAPQ filters*: proportion of reads that passed mapping quality filters. *Passed pre-WASP QC*: proportion of reads that passed the filtering stesp applied by WASP-indel before running the mappability filtering pipeline (number of variants on reads and allele combinations filters). *WASP mappability filter*: proportion of reads that passed the proper WASP-indel mappability filter, where reads are checked for comparable mappability across all alleles. *Duplicated removed*: proportion of reads after duplicates are removed. **D.** Percentage of reads overlapping indels that would have been filtered out by the original WASP pipeline ("Reads recovered by WASP-indel").

These reads are not discarded *a priori* by WASP-indel but instead are tested for mapping biases ("Mappability issues"). Only a fraction of reads is filtered out. Reads with "Allele combinations" tag are filtered *a priori* because they would require swapping in more than 64 allele combinations. Testing all allele combinations would be too computationally expensive. Reads with "Improper mapping" tag re filtered because of improper pair mapping.

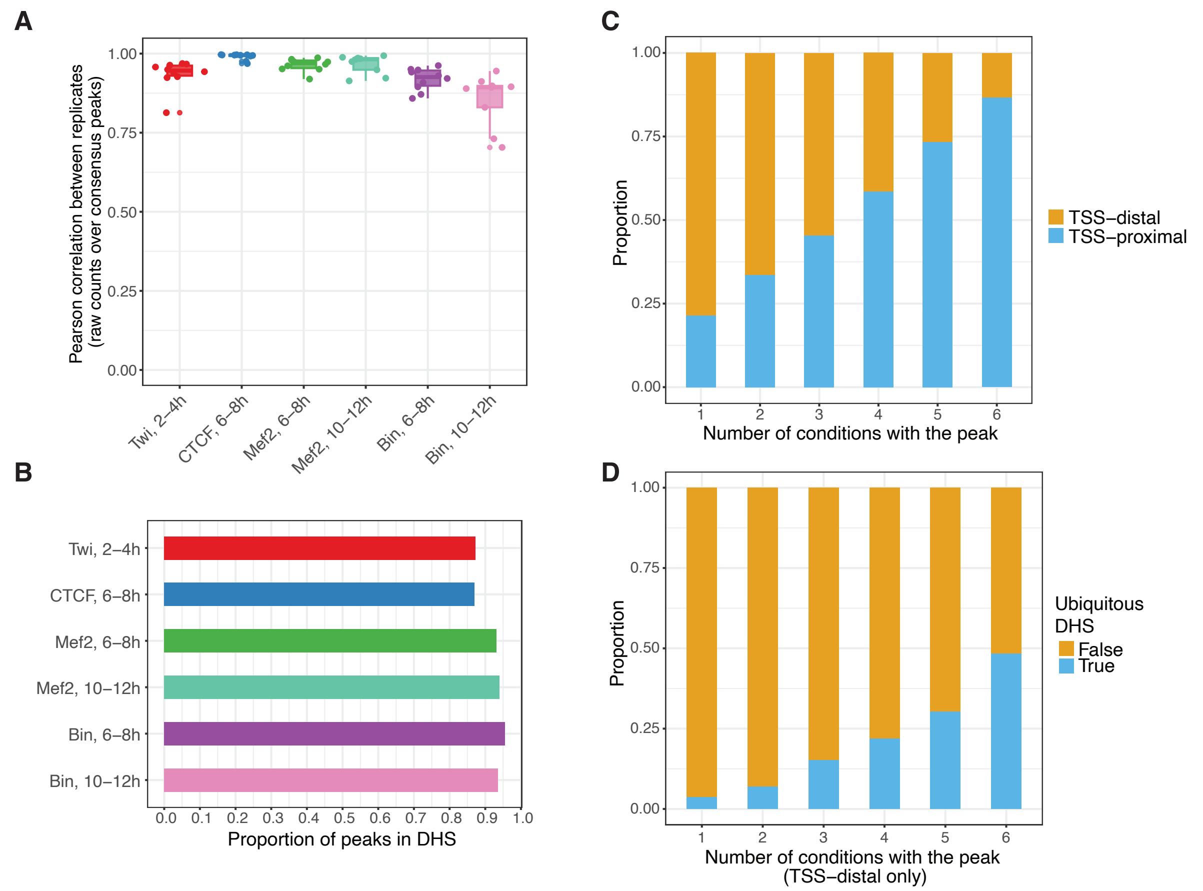

Supplemental Fig. 2: Properties of transcription factor ChIP peaks

A. Pearson correlation of raw signal under peaks between ChIP-seq biological replicates. **B.** Proportion of ChIP-seq peaks overlapping DHS peaks from *Reddington et al. 2020*. **C.** Proportion of TSS-Proximal and TSS-Distal co-bound sites, by the number of conditions (TF:time-point) in which TFs are bound. 1 = sites bound in a single condition, 6 = sites co-bound in all conditions included in this study. **D.** Proportion of ubiquitous DHS (from *Reddington et al. 2020)* overlapping co-bound sites, by the number of conditions (TF:time-point) in which TFs are bound.

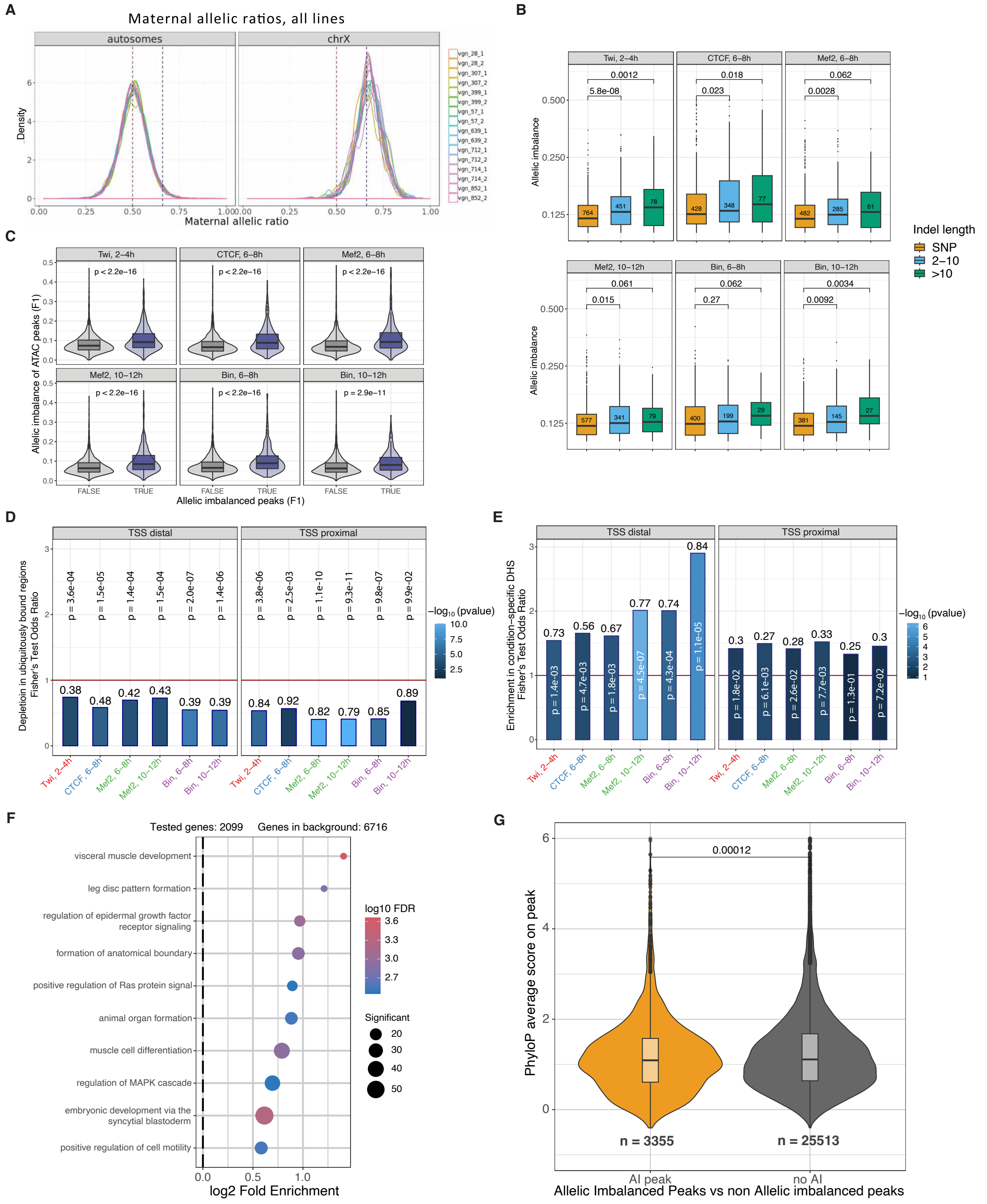

Supplemental Fig. 3: Properties of Allelic imbalance in transcription factor peaks

A. Distribution of Maternal Allelic Ratios ([Maternal] / [Maternal + Paternal]) for all transcription factor peaks in each F1 cross and biological replicate. Maternal Allelic Ratios are centered around 0.5 for autosomes and around 0.67 for Chromosome X. **B.** Effect size of Allelic Imbalance for SNPs, small indels (2-10bp) and larger indels (>10bp), Wilcoxon test p-value. **C.** Overlap between ChIP-seq Allelic imbalance (x-axis) and ATAC-seq allelic imbalance (y-axis) measured in the same F1 crosses in *Floc'hlay et al. 2021*. Allelic imbalanced peaks in all ChIP-seq conditions display higher imbalance in

ATAC-seq. **D.** Depletion (Fisher Test Odds Ratio) of allelic imbalanced peaks from ubiquitously bound peaks (defined as bound by >10 TFs in modERN dataset) across all conditions. Fisher's test odds ratios (imbalanced vs. non-imbalanced peaks) are plotted on y-axis. Numbers over the bars indicate the fraction of imbalanced peaks overlapping ubiquitously bound regions over all imbalanced peaks. Colour represents the Fisher's test p-value (-$\log_{10}$), the p-value is also reported on top of each bar. **E.** Enrichment (Fisher Test Odds Ratio) of allelic imbalanced peaks from condition specific DHS peaks (defined as DHS peaks found in only one condition in *Reddington et al. 2020*) across all conditions. Fisher's test odds ratios (imbalanced vs. non-imbalanced peaks) are plotted on y-axis. Numbers over the bars indicate the fraction of imbalanced peaks overlapping condition-specific DHS over all imbalanced peaks. Colour represents the Fisher's test p-value (-$\log_{10}$), the p-value is also reported on top of each bar. **F.** Gene Ontology enrichment of genes (ChIP-seq peaks are associated to the closest gene) comparing allelic imbalanced and non-imbalanced peaks. **G.** Distribution of phyloP 127 way scores on ChIP-seq peaks comparing allelic imbalance and non-imbalanced peaks (Wilcoxon two-tailed p-value).

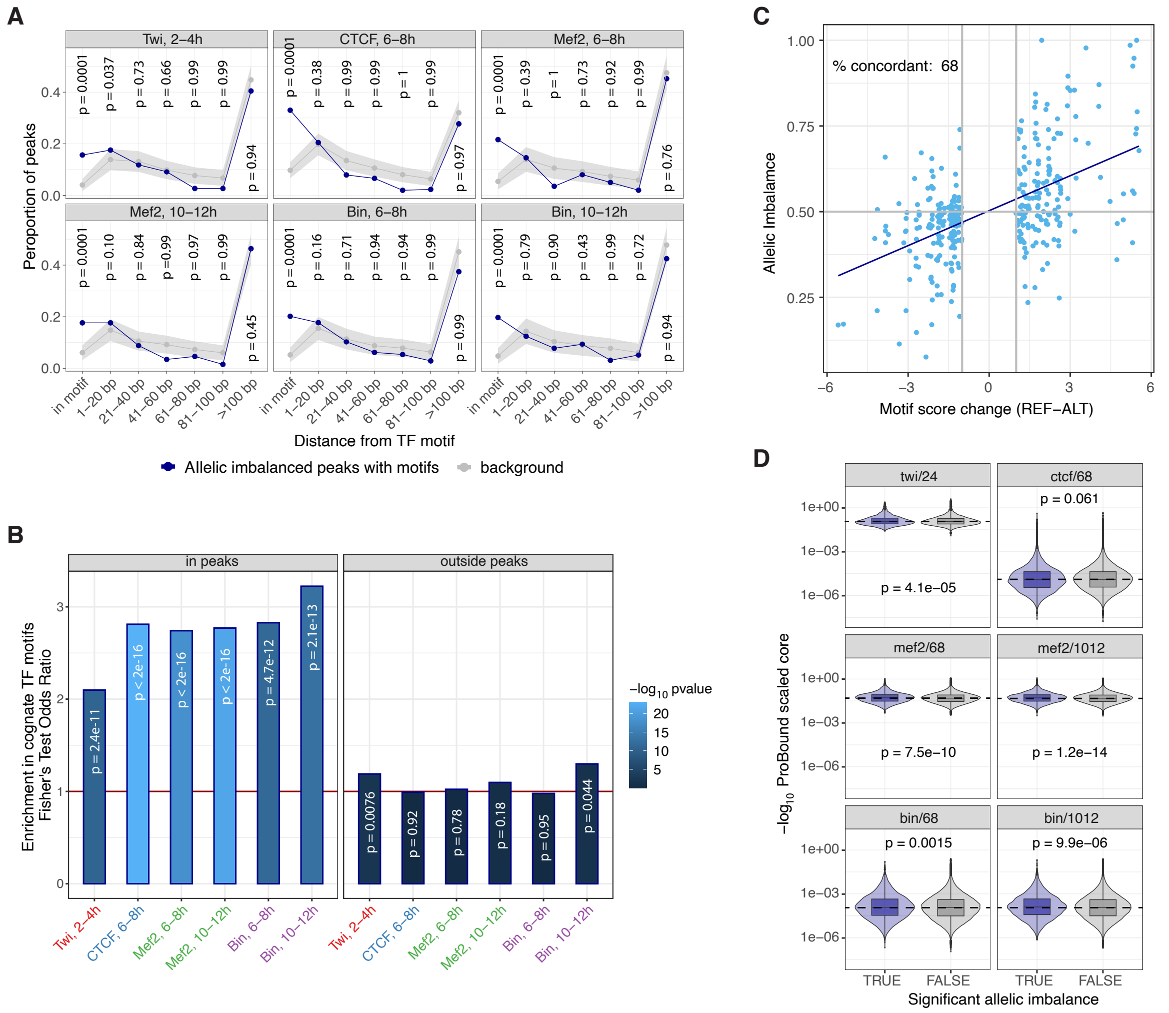

Supplemental Fig. 4: Overlap of AI associated significant variants in the TFs' cognate motif

A. Proportion of allelic imbalanced peaks with a significant variant at increasing distances of the transcription factor's own cognate motif (blue lines) compared to a background of non-imbalanced peaks (grey lines) across all conditions. The light grey bars correspond to 2 standard deviations of the background distribution. **B.** Enrichment (Fisher Test Odds Ratio) of allelic imbalanced variants overlapping the transcription factor's own cognate motif divided by variants within or outside the affected peak. Fisher Test p-values are reported inside each bar. **C.** Allelic imbalance for ChIP peaks (y-axis) vs motif score change (reference – alternative allele, x-axis) for significant variants inside transcription factor motifs. Only cases when the motif is called in both alleles are considered. Combined data for all conditions is shown. Only variants with an absolute motif score change > 1 are shown. Proportion of concordant variants (variants for which motif score change agrees with the direction of measured allelic imbalance) is indicated. **D.** ProBound transcription factors affinity scores on ±30bp sequences centered around the variant (REF allele). Variants are separated by being significantly imbalanced (TRUE) and non-significanlty imbalanced (FALSE). Wilcoxon test p-value comparing the distributions is reported.

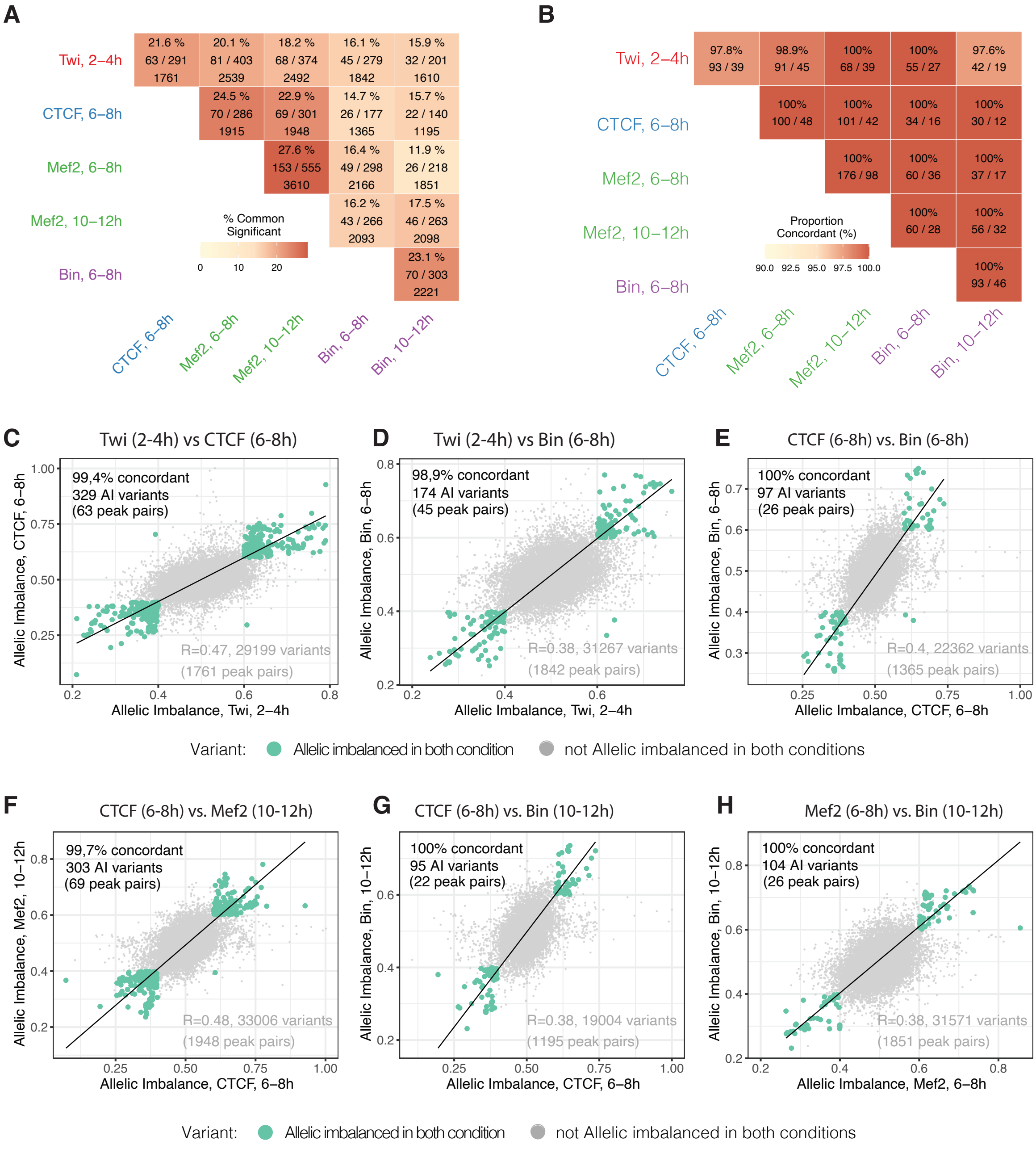

Supplemental Fig. 5: Concordant changes in AI for variants affecting two TFs
A. Percentage of concordant (same allelic imbalance direction) of variants in co-affected peaks for different conditions; percentage of concordant direction, number of significant shared variants / number of co-affected peaks are indicated. Only variants with the lowest CHT p-value per peak and condition are considered for each pair of co-affected peaks. **B.** Percentage of co-affected peaks over allelic imbalanced peaks in at least one condition. The top line shows the Jaccard coefficient (common and co-affected peaks / common peaks significantly imbalanced in at least one condition). The middle line outlines the number of coaffected peaks over the number of common peaks with allelic imbalance in at least one condition. The bottom line displays the total number of common peaks between the two conditions. The hetmap is coloured by the Jaccard coefficient. **C-H.** Correlation in allelic imbalance for variants affecting pairs (same as Fig. 4B-C) for conditions: (**C**) Twi at 2-4h vs CTCF at 6-8, (**D**) Twi at 2-4h vs Bin at 6-8h, (**E**) CTCF at 6-8h vs Bin at 6-8h, (**F**) CTCF at 6-8h vs Mef2 at 10-12h, (**G**) CTCF at 6-8h vs Bin at 10-12h, (**H**) Mef2 at 6-8h vs Bin at 10-12h. Each dot represents a variant (all

variants, imbalanced and not imbalanced, with unique allelic imbalance values per peaks overlapping between conditions are considered). Green dots represent variants that are allelic imbalanced in both conditions, grey dots represent variants that are not allelic imbalanced in both conditions (allelic imbalanced in one or no conditions). The percentage of concordance in allelic imbalance between conditions (same as in Fig. 4A), the number of variants and co-affected peaks are indicated for allelic imbalanced variants in both conditions are shown in black (top-left). Spearman correlation for all variants (green and grey dots), total number of variants and total number of peaks overlapping between conditions are shown in grey (bottom-right).

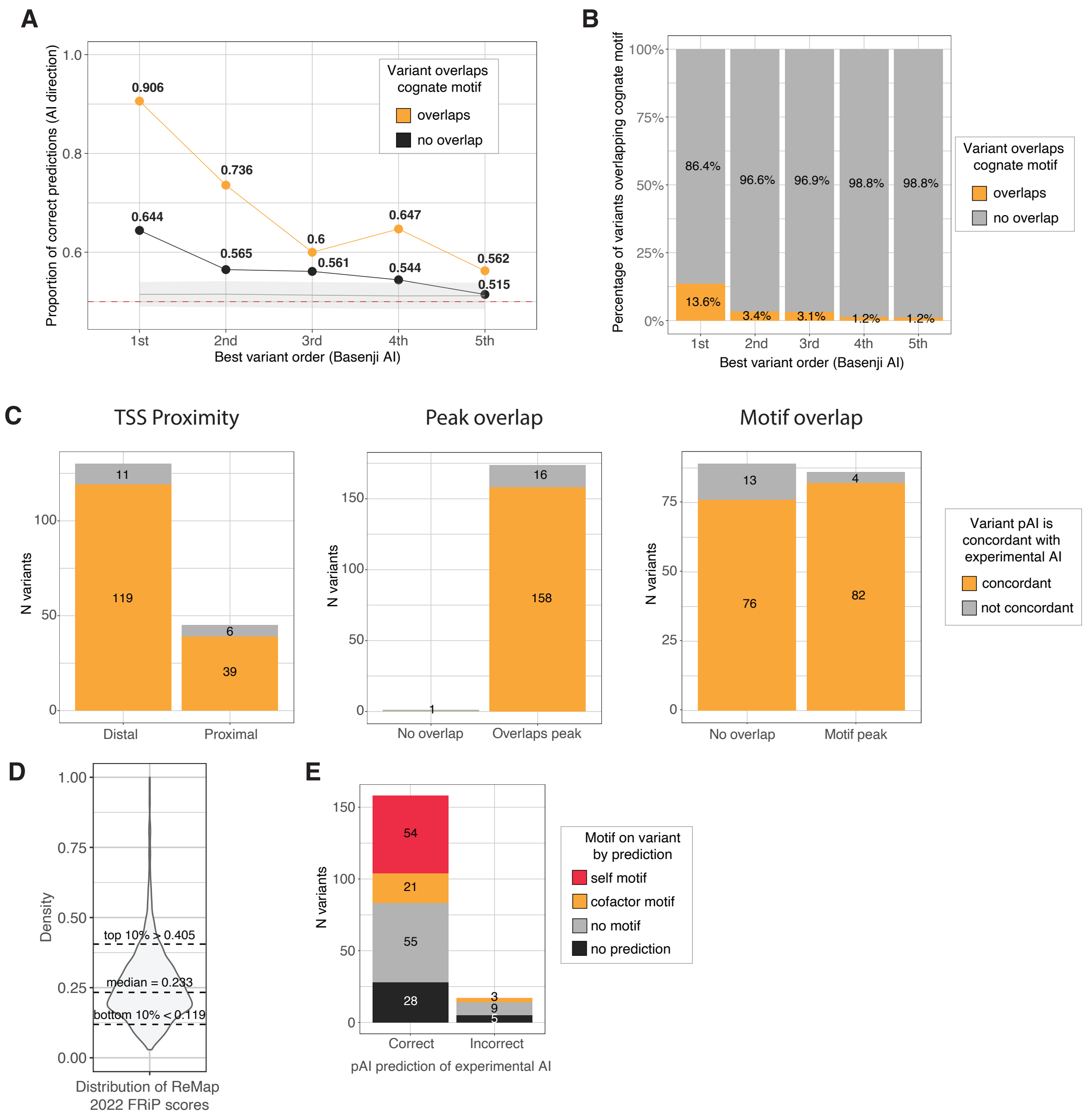

Supplemental Fig. 6: Basenji predictions of the impact of genetic variation on TF binding

A. Fraction of correct predictions (same direction of predicted imbalance (pAI) and experimentally measured allelic imbalance) for variants within the same peak, ranked by absolute pAI. This test includes all peaks associated with at least one variant with significant experimental imbalance (same data as Fig. 5C). Variants are stratified by overlapping the transcription factor's cognate motif (orange) or not overlapping the cognate motif (black). The grey line corresponds to 10,000 permutations of random variant ranking (background) and the light grey shadow represents 2 standard deviations. **B.** Percentage of variants ranked by pAI as in Fig. 5C that overlap the TF cognate motif. **C.** Counts of strong allelic imbalance and top-pAI variants whose Basenji prediction are concordant (orange) or not (grey) with the experimental allelic imbalance, divided by: TSS-Distal and TSS-Proximal peaks (*Left*), overlap with target peak overlap (*Center*), and overlap with cognate motif (*Right*). **D.** Distribution of ReMap2022 FRiP (Fraction of Reads in peaks) across 1205 *Drosophila* tracks used for Basenji training. Lines report the FRiP score for the 10th, 50th and 90th percentiles. **E.** Counts of strong allelic imbalance and top-pAI variants with the cognate motif (red), a potential co-factor motif (orange), no motif predicted (grey), non converging prediction (black) within ±75bp divided by correct and incorrect Basenji prediction.

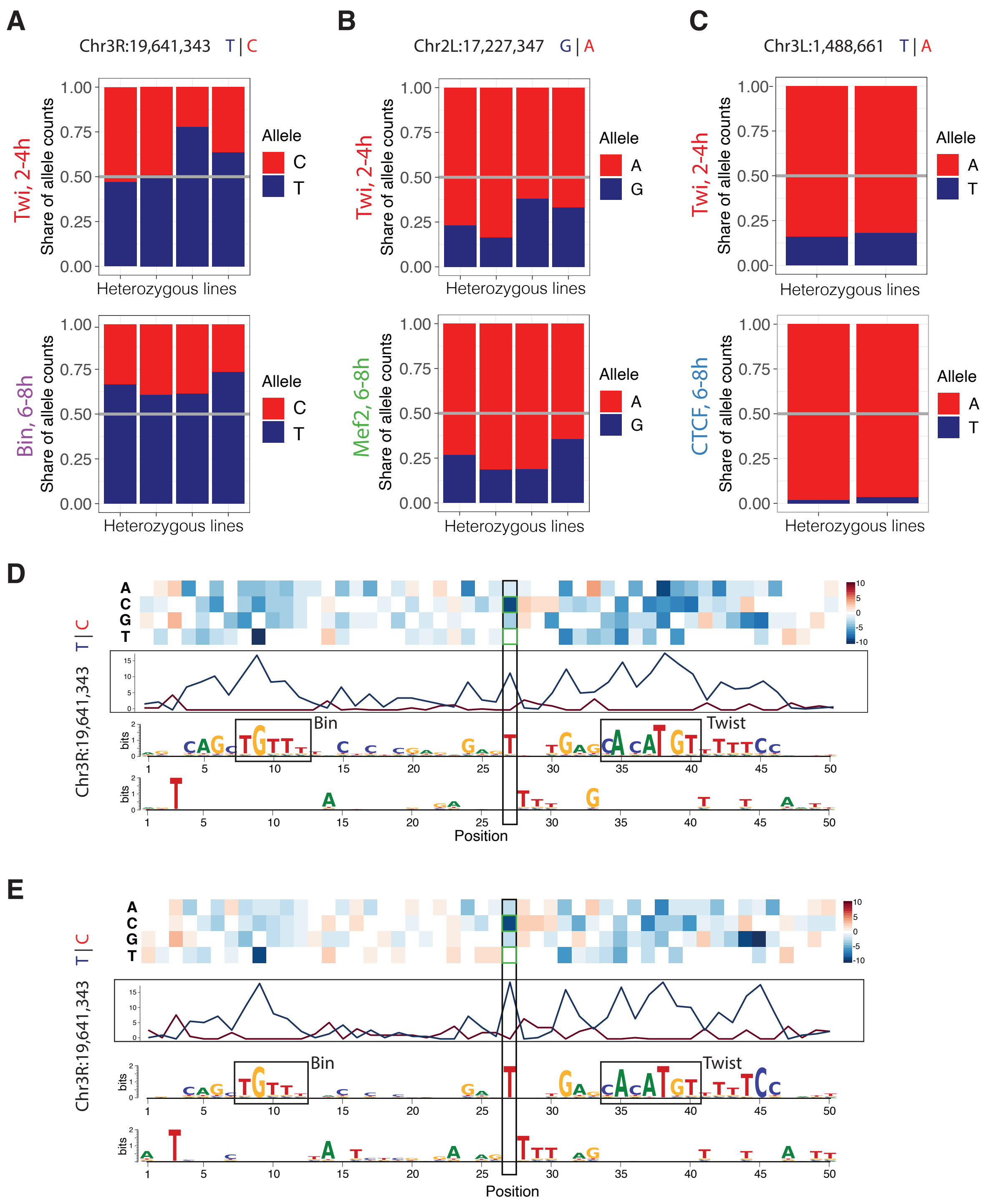

Supplemental Fig. 7: Basenji saturation mutagenesis around variants associated to multiple peaks

A-C. Experimental allelic imbalance for (**A**) Chr3R:19,641,343 in Twist at 2-4h and Biniou 6-8h, same variant reported in D-E; (**B**) Chr2L:17,227,347 in Twist at 2-4h and Mef2 6-8h; (**C**) Chr3L:1,488,661 in Twist at 2-4h and CTCF 6-8h. The four lines with a heterzygous allele for each variant are shown in A and B, and the two lines in C. In all cases the transcription factor's binding has a stronger allelic imbalance at the later time-point. As allelic imbalance is a ratio between maternal and paternal signal at the same time-point (both are proportional to total signal), it is independent of total peak signal suggesting that the stronger allelic imbalance at later time-points is biological, pointing to a downstream

effect across embryogenesis. **D-E.** Saturation mutagenesis around variant Chr3R:19,641,343 for Twist at 2-4h (**D**) and Biniou at 6-8h (**E**), same *locus* as in A. This variant is positioned between Twist and Biniou motifs and the saturation score recovers them. *Top*: Saturation mutagenesis scores (blue: negative values, red: positive values). Negative values indicate that swapping the bases will lead to lower TF binding, while positive values indicate that it will increase TF binding. *Center*: Importance scores given by the sum of negative (blue line) and positive (red line) saturation scores per base-pair. *Bottom:* Reconstructed motifs based on the reference sequence and negative (top) and positive (bottom) importance scores.

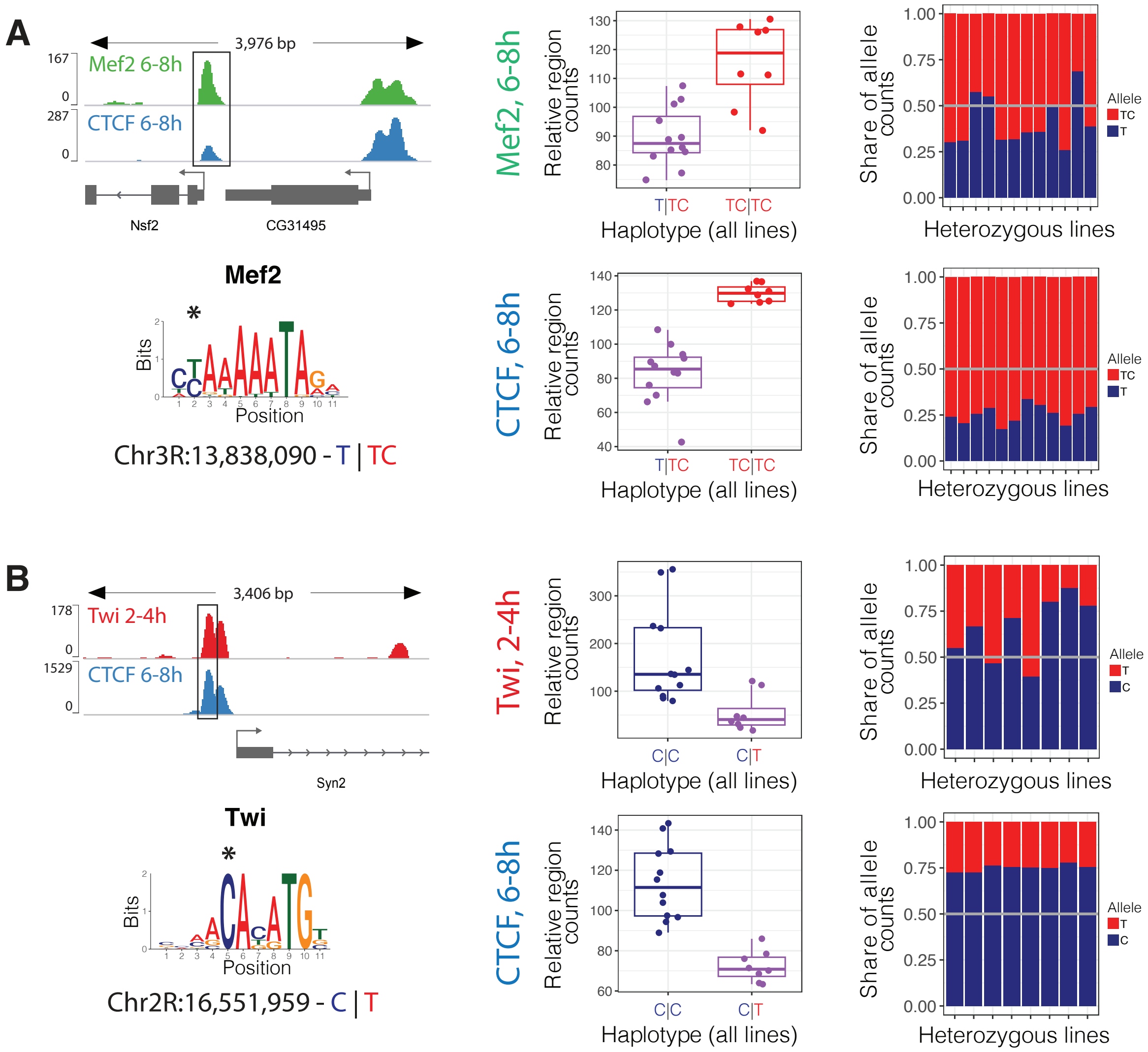

Supplemental Fig. 8: Examples of allelic imbalance in a TF motif impacting two TF binding events

A. *Left:* Variant Chr3R:13,838,090 is a T to TC short indel that overlaps the Mef2 motif and co-affects Mef2 and CTCF binding at 6-8h. The TC (ALT, red) allele corresponds to the Mef2 motif, while the T (REF, blue) allele corresponds to a deletion of the first A in the motif (asterisk). *Center:* Only the T|TC and the TC|TC alleles are found across the F1 lines. Lines harboring the T|TC allele show lower signal for both Mef2 and CTCF compared to the TC|TC lines. Y-axis = relative peak counts (read counts per sample, per peak normalised by library size), to account for read depth. *Right:* Both Mef2 and CTCF display higher binding for the TC allele in the heterozygous lines (corresponding to the purple samples in the boxplot). **B.** *Left:* Variant Chr2R:16,551,959 is a C to T SNP that overlaps the Twist motif and co-affects Twist binding at 2-4h and CTCF binding at 6-8h. The C (REF, blue) allele corresponds to the Twist motif, while the T (ALT, red) allele corresponds to a substitution of the first C in the motif (asterisk). *Center:* Only the C|C and the C|T alleles are found across the F1 lines. Lines harboring the C|C allele show higher signal for both Twist and CTCF compared to the C|T lines. Y-axis = relative peak counts (normalised by library size). *Right:* Both Twist and CTCF display higher binding for the C allele globally in the heterozygous lines (corresponding to the purple samples in the boxplot).

SUPPLEMENTAL TABLE LEGENDS

Supplemental Table S1: ChIP-seq samples information

Table describing the F1 ChIP-seq samples by replicates, time-points, antibodies and the respective parental lines. Total number of reads is also reported.

Supplemental Table S2: Consensus peaks set and IDR pseudoreplicates

ChIP-seq consensus peak set across transcription factors and time-points. Peak activity per condition is binarized (0: peak is not present; 1: peak is present). The second sheet reports the pooled and self pseudoreplicates IDR peaks numbers as a metric for peak calling reproducibility.

Supplemental Table S3: Motifs enrichment at allelic imbalanced peaks

Cis-BP and selected motifs enrichment on non-significant, weakly significant and strongly significant allelic imbalanced peaks across the 6 conditions.

Supplemental Table S4: Coaffected peaks linked to the same variant associated to motif disruption

List of variants linked to the same peak across two conditions where the variant also impacts the motif of at least one of the two transcription factors. The conditions, peak IDs, variant distance to the closest TF motif and allelic imbalance are reported.

Supplemental Table S5: ReMap 2022 full dataset metatable

List of all ReMap 2022 samples included in Basenji's training. Samples annotations have been obtained from ReMap 2022. Corresponding samples metadata and input files are reported (if present). Samples were grouped by experiment ID for Basenji's training.

Supplemental Table S6: Saturation mutagenesis motif discovery

Motifs recovered by saturation mutagenesis on 175 strong predicted Allelic Imbalanced variants.

SUPPLEMENTAL NOTES

Note 1: Twist was discovered in *Drosophila* for its loss of function mutant phenotype, which is a block in the gastrulation of the presumptive mesoderm that occurs at stage 6 (Baylies and Bate 1996), which is covered by the two-hour window profiled in this study (2-4hr). A number of genetic studies showed that Twist directly regulates the expression of several key transcription factors (TFs) at 2-4h that are essential for the specification of the mesoderm into different muscle lineages – including Tinman (Khoueiry et al. 2017) and Mef2 (Cripps et al. 1998). This is the reason why this time window was selected for Twist, as it's the most critical time window for Twist's function during embryogenesis. We recently confirmed this using an optogenetic depletion approach to deplete Twist protein from the nucleus at different embryonic stages – first allowing the embryos to gastrulae and then depleting twist from the mesoderm (Kögler et al. 2021). This revealed that Twist is required for embryogenesis up until stage 9/10 – but has lower impact at stage 11 (corresponding to the 6-8h window used for the other TFs). Given all of those reasons, we chose the 2-4h time-window for these experiments, as there is no one embryonic window that covers the peak of all four TFs requirement during embryogenesis.

Note 2: Approximately 88% of TF peaks have at least one genetic variant within ±2.5kb of the peak summit and 82% have more than 20 variants. This is due to the extensive genetic diversity among these inbred wild-isolates, which have a median of 1 SNP per 227-236bp. It is also uncovered here due to how deeply we sequenced the parental genotypes that we are working with, with 74-125x sequencing coverage for each line. This is feasible given the size of the *Drosophila* genome, and means that we know the full complement of genetic variants (SNPs and indels). This is generally not the case with studies in other species, especially humans, as such a sequencing depth genome-wide is cost prohibitive, and therefore often only achieved on portions of the genome e.g. exome sequencing. Typically, there is more shallow sequencing genome-wide, which will have a lot of 'hidden' LD. *Drosophila* also has a higher variant density compared to mammals making it more likely that variants are very close on the linear genome. This results in blocks of LD being relatively well contained, for example, allelic imbalanced transcription factor peaks are linked to a median of 9 variants. In order to uncover the causal variant, we used deep learning to infer the causal variant and provide insight into the variant's mechanisms.

Note 3: The overarching aim of this study was to quantify the impact of genetic variation on transcription factor (TF) binding, uncovering both direct (e.g. genetic variants in the TF's own cognate motif) and indirect genetic effects. The latter could be caused by disruption of cooperative TF binding (direct contact), nucleosome binding, or cooperativity spanning larger distances over multiple nucleosomes, where the motifs are in proximity at the same face of the DNA after wrapping round the nucleosome. Zooming out, enhancers (or different regulatory elements) can also have cooperative effects on each other's activity (e.g. variable Tomatin modules; (Waszak et al. 2015)) or the recently identified facilitator elements (Blayney et al. 2023).

To capture the contribution of indirect genetic effects, we included variants outside the TF ChIP peaks. We chose ±2.5kb around the peak summit to strike a balance between sensitivity to capture an indirect event, multiple testing (since each variant is tested independently) and the high density of linked variants (which makes it complicated to pinpoint the causal variant within linkage blocks), and the compact nature of the *Drosophila* genome (which often has many enhancers (or DHS/ATAC peaks) clustered together within 2-3kb). Our analysis suggests that indirect genetic effects are widespread (35-55% of imbalanced peaks had the most significant associated variant outside the peak).

Note 4: Permuting genotype information revealed some deviation from the uniform p-value distribution (CHT permuted, Fig. 2A). This may result from the close relatedness among lines (all F1s sharing the same mother). To assess this, we performed random permutation of the genotypes, which identified 0.28-2.0% potential false associations within the FDR range, indicating that relatedness does not lead to an excess of false associations in this study.

Note 5: Although CTCF tracks are overrepresented in the training data (2% of all tracks, compared to ~0.2% for the other 3 factors), when looking at the prediction metrics (that indicate how well Basenji can predict the coverage of each track in the test dataset), it shows that the model can on average predict the coverage of Mef2, Bin and Twist better than the one of CTCF.:

- CTCF: 0.52613
- Mef2: 0.77526
- Bin: 0.77476
- Twi: 0.73360

A higher proportion of signal coming from ReMap tracks, where Basenji performs worse (Fig. 5B), could perhaps explain the lower median Person r for CTCF.

We note that imbalanced training sets are unfortunately the norm in machine learning in general. The large overlap between different transcription factors binding mitigates the imbalance somewhat since features from each transcription factor can also be learned from other transcription factors that have (at least partial) overlapping binding. In our case, the imbalanced nature of the training set does not lead to a single transcription factor (or experiment) dominating the training set. This means that it will not bias our predictions towards a single factor (or experiment), but that we might have poorer predictions for some TFs compared to others based on how much they are represented. Thank you for pointing this out.

Note 6: 78 out of 175 peaks are called in 2 or more conditions so they can be potentially allelic imbalanced in more than one condition. All of them are significantly allelic imbalanced in at least one condition (by definition of strong pAI) making the enrichment method comparable to Fig. S5H. 7 of the 78 (9.0%) are strong pAI in more than one condition. It is difficult to have an expectation if this is lower than we would expect. It is lower than observed when looking at all variants (Fig. S5H), but here we are considering a small subset of all associations (significant experimental allelic imbalance in both cases and strong predicted imbalance (pAI) in both cases), which reduces the overlap dramatically. Out of these 7 cases, by definition, all are significant for two conditions both in experimental allelic imbalance and in predicted imbalance (strong pAI (points shown in Fig. 5E)). The peaks are also overlapping between conditions for the 7 variants. This was not forced and it's again another example of reproducibility. For further investigation, all examples can be explored using the Shiny app (http://furlonglab.embl.de/data). The variant IDs are:

- Chr2L:17227347
- Chr2L:5783401
- Chr2R:14224310
- Chr3L:1488661
- Chr3R:19641343
- Chr3R:30735251
- Chr3R:8689135

These are great examples of reproducibility. Moreover, the concordance in the direction of change in both the experimental and predicted AI for all cases is another indirect proof of the reproducibility of our results.

SUPPLEMENTAL METHODS

Drosophila genetics and embryo collection

To obtain F1 hybrid embryos, males from eight genetically distinct inbred lines from the DGRP collection (MacKay et al. 2012) were crossed to females from a "virginizer" (VGN) line. The VGN line contains a heat-shock-inducible pro-apoptotic gene (*hid*) on the Y Chromosome (Starz-Gaiano et al. 2001) of a laboratory reference strain (*w1118*) that kills all male embryos after a 37°C heat-shock.

Freshly hatched adults were placed in embryo collection containers with standard apple cap plates. For ChIP-seq experiments, following three 1 h pre-lays, the flies were allowed to lay embryos for 2h. The embryos were then aged for 2h, 6h or 10h to reach the developmental intervals 2-4h, 6-8h or 10-12h. Embryos were collected and dechorionated using 50% bleach, and washed with deionized water (dH2O) and PBT (phosphate buffered saline (PBS) containing 0.1% Triton X-100). Dechorionated embryos were crosslinked in 1.8% formaldehyde for 15 min at room temperature. Fixation was stopped by the addition of PBT + Glycine 125mM and embryos were washed with PBT. After drying the embryos on tissue, they were snap-frozen in liquid nitrogen and store at -80˚C until use.

ChIP-seq on *Drosophila* embryos

ChIP-seq was performed as described in (Bonn et al. 2012). For each ChIP, 2.5 ug of chromatin was used for all conditions, including input, using antibodies directed against Twist (Sandmann et al. 2007), Mef2 (Sandmann et al. 2006) and Biniou (Jakobsen et al. 2007) generated in our lab, and against CTCF from R. Reinkawitz's lab. Antibodies were incubated overnight with chromatin in RIPA buffer in a total volume of 900 ml using 3 ml of rabbit anti-Mef2, 1 ml guinea pig anti-Twist, 1 ml rabbit anti-Biniou, and 1 ml of rabbit anti-CTCF for all lines. The next day 25 ml of magnetic protein A/G beads (Dynabeads, Invitrogen, 10002D and 10004D) were washed with 1ml of RIPA buffer and added to the IPs for an additional 3 hour incubation on the rotating wheel at 4°C. The ChIPs were then washed for 10 min on the rotating wheel with 1x 1 ml RIPA, 4x 1 ml RIPA-500 (500 mM NaCl, 10 mM Tris-HCl pH 8.0, 1 mM EDTA, 1% Triton X-100, 0.1% SDS, 0.1% Na-deoxycholate), 1x 1 ml LiCL buffer (250 mM LiCl, 10 mM Tris-HCl pH 8.0, 1 mM EDTA, 0.5% IGEPAL CA-630 CA-630, 0.5 % Na-deoxycholate) and 2x 1 ml TE buffer (10 mM Tris pH 8.0, 1 mM EDTA) in the cold room. The chromatin was then RNase-treated and reverse cross-linked as described for the quality check of the chromatin.

A molecular barcoded ChIP-seq library was prepared with all ChIPed-DNA obtained using the NEXTflex™ qRNA-seq™ Kit v2 (HiSS Diagnostics, 520999-03). The quality of the libraries was assessed on a Bioanalyzer (Agilent), and libraries displayed a peak around 350-600 bp. For each ChIP, at least two completely independent biological replicates were performed. ChIP-seq libraries were paired-end sequenced with 81bp PE for ChIP samples and 151bp PE for ChIP inputs using an Illumina NextSeq 500 platform at the EMBL Genomics Core Facility.

Genomic DNA sequencing of parental lines

We collected 10 adult flies from each of the parental lines used in this study were collected and snap frozen: DGRP-28, DGRP-307, DGRP-399, DGRP-57, DGRP-639, DGRP-712, DGRP-714, DGRP-852 and VGN. To extract the genomic DNA, the flies were crushed with a handheld pestle until powdered, while kept frozen into liquid nitrogen. 500 µl buffer A (Tris-HCl pH 7.5 100 mM, EDTA pH 8.0 100mM, NaCl 100mM, SDS 1%) were added and mixed with the powdered flies. 20 µl of PK Proteinase K (PK) 20 mg/ml were added and the samples incubated for 3h at 65°C. PK was inactivated by incubation at 95°C for 10 min. The samples were then treated with 1 µl RNAse A 10 mg/ml and incubated for 30 min at 37°C. DNA was extracted with 500 µl phenol/chlorophorm/IAA, followed by 500 µl chlorophorm. The aqueous phase was transferred to a new tube and 3 volumes of 100% ethanol were added. The pellet was washed with 1 ml 70% EtOH and air-dried and resuspended in 25 µl 10mM Tris-HCl. 2 µg of gDNA per sample were diluted in 100 µl of TE buffer. The gDNA was sonicated with a Bioruptor Pico using 6 cycles of 30s ON and 90s OFF. 1 µg of sheared gDNA was used for library preparation with the NEBNext Ultra II DNA library kit. We enriched for fragments of 300-400bp of length and amplified the library with 3 PCR cycles.

ChIP-seq read mapping and peak calling

FASTQ files with ChIP-seq reads were pre-processed by removing unique molecular identifiers (UMIs) using JE v.1.2 (Girardot et al. 2016) with parameters *LEN=8 BPOS=BOTH ADD=TRUE SAME_HEADERS=TRUE XT=1 ZT=0 RCHAR=':'* and removing adaptor readthrough with skewer v0.2.2 (Jiang *et al*, 2014) with parameters *-m pe -z -x 'NNNNNNNNNAGATCGGAAGAGCACACGTCTGAACTCCAGTCACNNNNNNNNATCTCG TATGCCGTCTTCTGCTTG' -y*

'NNNNNNNNNAGATCGGAAGAGCGTCGTGTAGGGAAAGAGTGTAGATCTCGGTGGTCG CCGTATCATT'.

Low quality bases were removed with seqtk trimfq v1.2 (https://github.com/lh3/seqtk) with default parameters. Reads were mapped to the reference dm6 genome with BWA v0.7.15 (Li and Durbin 2010) using BWA-MEM with option `-M`. BAM files were sorted and indexed with Picard SortSam (Broad Institute, 2015). Reads were filtered requiring a minimum mapping quality of 10. One input sample (VGN-DGRP307 at 2-4h) was removed due to failed QC. For the peak calling, input samples were merged across all lines per time-point (see below).

ChIP Peak calling and Irreproducibility Discovery Rate (IDR) analysis:

We implemented an IDR pipeline (Li et al. 2011) as recommended in ENCODE guidelines (Landt et al. 2012). ChIP peaks were called using MACS2 (Zhang et al. 2008) with relaxed p-value threshold (0.5) for each replicate/pseudoreplicate '-p 0.5 --format BAMPE --gsize 142573017'. Input files used in peak calling were merged per time-point (across all lines) using SAMtools merge v.1.9 (Li et al. 2009). IDR tool v2.0.4.2 was used (https://github.com/nboley/idr). The number of peaks at IDR 1%, 2% and 5% (global IDR value) were recorded (Supplemental Table S2) from the following steps:

- Between true biological replicates (Nt)
- Between pooled pseudoreplicates (Np)
- Between self-pseudoreplicates of biological replicate 1 (N1)
- Between self-pseudoreplicates of biological replicate 2 (N2).

The majority of samples showed high reproducibility and satisfied the following quality criteria suggested for the IDR analysis: (1) Nt and Np within a factor of 2 and (2) N1 and N2 within a factor of 2 (Supplemental Table S2). At IDR 1%, two samples for one line (VGN-DGRP714 for Mef2 and Bin at 10-12h) showed a very low number of reproducible peaks between replicates (Nt=130 and 51, respectively) due to a failed replicate 1 (N1=354 and 117, respectively), and were therefore excluded from all downstream analysis. Based on this QC analysis, we included reproducible peaks at IDR 1%, and required a peak to be present in at least three fly lines to be included in the consensus peak sets (see below).

Defining consensus peak sets: Consensus peak sets were constructed using DiffBind package in R (Ross-Innes et al. 2012). For each condition (TF + time-point), we used reproducible peaks between true replicates ('conservative' peak set) at 1% IDR in each line.

The consensus set was produced with dba.count function using TMM normalization (score=DBA_SCORE_TMM_READS_FULL), fragment size from BAM files (fragmentSize = 0) and control reads were normalized by relative library size (default). Only peaks present in at least three lines were considered (minOverlap = 6 since we have 2 replicates for each line). For the resulting consensus set, summits were recalculated based on read coverage, and consensus peaks were resized to 500 bp around the new summits (summits=250).

Allelic imbalanced peak Gene Ontology enrichment and conservation:

ChIP-Seq peaks with significant allelic imbalance were tested for Gene Ontology enrichment compared to non-imbalanced peaks. Allelic imbalanced and non-imbalanced peaks were annotated for TSS-proximity and associated to the closest gene with the R package ChIPseeker (Pollard et al. 2010). The Gene Ontology enrichment was performed with the R package topGO using the "Biological Process" terms and the weight algorithm which accounts for GO terms topology. Significantly enriched terms (FDR < 0.01) with more than 10 and less than 500 genes associated with them are reported in Supplemental Fig. S3F.

To test for sequence conservation, we downloaded the phyloP 127 way conservation scores (Pollard et al. 2010) from UCSC and overlapped them with the ChIP-seq peaks. Each peak was associated to the weighted length average of the phyloP scores overlapping it.

Motif analysis:

De novo motif discovery and central enrichment was performed on the top 1000 peaks from the consensus peak sets from each condition (ranked by average TMM scores across all samples) using MEME-ChIP v 4.11.3 (Bailey et al. 2009) with second order of the Markov background model (-order 2). This was complemented by scanning for known TF motifs in the consensus peak sets (separately for each condition) and 30 bp intervals centred on all quantified variants (both reference and alternative allele) using FIMO 4.11.3 (Bailey et al. 2009) on both strands (default) with nucleotide frequencies as background (calculated with fasta-get-markov v. 4.11.3, from MEME suite). We used optimized PWMs for Twist, Mef2, and Biniou (Zinzen et al. 2009), while all other PWMs were downloaded from Cis-BP database (Weirauch et al. 2015) version from 05.03.2019.

For all quantified variants, the distance to the nearest motif was recorded. To quantify the distribution of AI peaks by variant to motif distance (Fig. 3A), we selected the closest

significant variant to the cognate TFBS for each peak. To construct a background set for each condition (Supplemental Fig S4A), we used the following procedure: (1) from non-AI peaks with motifs, randomly select the same number of peaks as in AI peak set, (2) from the selected peak set select the number of associated variants equal to the number of significant variants, (3) for each peak, record minimum distance of variants to motif. This procedure was repeated 100 times, and the average number of peaks was reported for each distance interval.

For reconstructing motif logos from allele-specific binding (Fig. 3F-G), we used all significant SNPs with AI > 0.1 overlapping motifs of corresponding TFs. For each position in the motif, we selected nucleotides that were preferably bound by the TF based on allele-specific information, e.g. selecting reference allele if $\alpha > \beta$ for the considered SNP and alternative allele otherwise. For each TF, we constructed positional frequency matrixes (PFM) based on the numbers of nucleotides preferentially bound at each position affected by variants. Resulting PFMs were then used as input to make PWM and seqLogo functions from the seqLogo package (Bembom and Ivanek 2024) in R with default parameters.

To calculate ProBound affinity scores (Rube et al. 2022) we reconstructed the reference sequence around each variant (30bp upstream and downstream) with BEDTools getfasta (Quinlan and Hall 2010). The relative affinity for each sequence was then obtained by running the command "java -cp ProBound-jar-with-dependencies.jar proBoundTools/App -c 'loadMotifCentralModel().addNScoring().inputTXT().bindingModeScores()'". We used available models from MotifCentral (Rube et al. 2022) for the four Transcription Factors in this study (in particular CTCF: 12715, Mef2: 13466, Bin: 15352, Twi: 15424).

ReMap 2022 coverage tracks:

The mapped BAM files were processed using the ReMap analysis pipeline (Hammal et al. 2022), provided by Fayrouz Hamal and Benoît Ballester. According to the database annotation we were able to match 570 out of 1205 ChIP-seq samples to at least one corresponding ChIP input (same publication, conditions and sample of origin). In cases where multiple ChIP inputs were associated to the same sample, the BAM files were merged with SAMtools merge (Li et al. 2009). All coverage tracks (ChIP samples and inputs) were generated using BAMCoverage (Ramírez et al. 2016) with the following options "--binSize 10 --normalizeUsing RPGC --effectiveGenomeSize 125464728 --ignoreForNormalization chrX chrM chrY" with option "--extendReads 0" for single-end reads (no reads extention) and "--extendReads" for paired-end

reads (reads were extended to match the fragment size). Samples with a corresponding ChIP input were input subtracted with bigWigCompare (Ramírez et al. 2016) with options "--binSize 10 --operation subtract". Replicates were merged with bigWigAverage. All bigWigs are available for browsing and download at: http://furlonglab.embl.de/data.

BIBLIOGRAPHY

Bailey TL, Boden M, Buske FA, Frith M, Grant CE, Clementi L, Ren J, Li WW, Noble WS. 2009. MEME SUITE: tools for motif discovery and searching. *Nucleic Acids Res* **37**: W202-8.

Baylies MK, Bate M. 1996. twist: A Myogenic Switch in Drosophila. *Science (1979)* **272**: 1481–1484. https://www.science.org/doi/10.1126/science.272.5267.1481 (Accessed November 6, 2024).

Bembom O, Ivanek R. 2024. seqLogo: Sequence logos for DNA sequence alignments. *R package version 1700*.

Blayney JW, Francis H, Rampasekova A, Camellato B, Mitchell L, Stolper R, Cornell L, Babbs C, Boeke JD, Higgs DR, et al. 2023. Super-enhancers include classical enhancers and facilitators to fully activate gene expression. *Cell* **186**: 5826-5839.e18. https://pubmed.ncbi.nlm.nih.gov/38101409/ (Accessed January 24, 2025).

Bonn S, Zinzen RP, Girardot C, Gustafson EH, Perez-Gonzalez A, Delhomme N, Ghavi-Helm Y, Wilczyåski B, Riddell A, Furlong EEM. 2012. Tissue-specific analysis of chromatin state identifies temporal signatures of enhancer activity during embryonic development. *Nat Genet* **44**: 148–156.

Cripps RM, Black BL, Zhao B, Lien CL, Schulz RA, Olson EN. 1998. The myogenic regulatory gene Mef2 is a direct target for transcriptional activation by Twist during Drosophila myogenesis. *Genes Dev* **12**: 422–434. https://pubmed.ncbi.nlm.nih.gov/9450935/ (Accessed January 24, 2025).

Girardot C, Scholtalbers J, Sauer S, Su SY, Furlong EEM. 2016. Je, a versatile suite to handle multiplexed NGS libraries with unique molecular identifiers. *BMC Bioinformatics* **17**: 1–6. https://bmcbioinformatics.biomedcentral.com/articles/10.1186/s12859-016-1284-2 (Accessed May 3, 2024).

Hammal F, De Langen P, Bergon A, Lopez F, Ballester B. 2022. ReMap 2022: a database of Human, Mouse, Drosophila and Arabidopsis regulatory regions from an integrative analysis of DNA-binding sequencing experiments. *Nucleic Acids Res* **50**: D316–D325. https://dx.doi.org/10.1093/nar/gkab996 (Accessed December 11, 2023).

Jakobsen JS, Braun M, Astorga J, Gustafson EH, Sandmann T, Karzynski M, Carlsson P, Furlong EEM. 2007. Temporal ChIP-on-chip reveals Biniou as a universal regulator of the visceral muscle transcriptional network. *Genes Dev* **21**: 2448–2460. http://genesdev.cshlp.org/content/21/19/2448.full (Accessed December 4, 2023).

Khoueiry P, Girardot C, Ciglar L, Peng PC, Hilary Gustafson E, Sinha S, Furlong EEM. 2017. Uncoupling evolutionary changes in DNA sequence, transcription factor occupancy and enhancer activity. *Elife* **6**.

Kögler AC, Kherdjemil Y, Bender K, Rabinowitz A, Marco-Ferreres R, Furlong EEM. 2021. Extremely rapid and reversible optogenetic perturbation of nuclear proteins in living embryos. *Dev Cell* **56**: 2348-2363.e8. https://pubmed.ncbi.nlm.nih.gov/34363757/ (Accessed January 24, 2025).

Landt SG, Marinov GK, Kundaje A, Kheradpour P, Pauli F, Batzoglou S, Bernstein BE, Bickel P, Brown JB, Cayting P, et al. 2012. ChIP-seq guidelines and practices of the ENCODE and modENCODE consortia. *Genome Res* **22**: 1813–1831.

Li H, Durbin R. 2010. Fast and accurate long-read alignment with Burrows-Wheeler transform. *Bioinformatics* **26**: 589–595.

Li H, Handsaker B, Wysoker A, Fennell T, Ruan J, Homer N, Marth G, Abecasis G, Durbin R. 2009. The Sequence Alignment/Map format and SAMtools. *Bioinformatics* **25**: 2078–2079. https://pubmed.ncbi.nlm.nih.gov/19505943/ (Accessed May 3, 2024).

Li Q, Brown JB, Huang H, Bickel PJ. 2011. Measuring reproducibility of high-throughput experiments. *Ann Appl Stat* **5**: 1752–1779.

MacKay TFC, Richards S, Stone EA, Barbadilla A, Ayroles JF, Zhu D, Casillas S, Han Y, Magwire MM, Cridland JM, et al. 2012. The Drosophila melanogaster Genetic Reference Panel. *Nature* **482**: 173–178.

Pollard KS, Hubisz MJ, Rosenbloom KR, Siepel A. 2010. Detection of nonneutral substitution rates on mammalian phylogenies. *Genome Res* **20**: 110–121. https://genome.cshlp.org/content/20/1/110.full (Accessed October 25, 2024).

Quinlan AR, Hall IM. 2010. BEDTools: a flexible suite of utilities for comparing genomic features. *Bioinformatics* **26**: 841–842. https://dx.doi.org/10.1093/bioinformatics/btq033 (Accessed November 19, 2024).

Ramírez F, Ryan DP, Grüning B, Bhardwaj V, Kilpert F, Richter AS, Heyne S, Dündar F, Manke T. 2016. deepTools2: a next generation web server for deep-sequencing data analysis. *Nucleic Acids Res* **44**: W160–W165.

Ross-Innes CS, Stark R, Teschendorff AE, Holmes KA, Ali HR, Dunning MJ, Brown GD, Gojis O, Ellis IO, Green AR, et al. 2012. Differential oestrogen receptor binding is associated with clinical outcome in breast cancer. *Nature* **481**: 389–393.

Rube HT, Rastogi C, Feng S, Kribelbauer JF, Li A, Becerra B, Melo LAN, Do BV, Li X, Adam HH, et al. 2022. Prediction of protein–ligand binding affinity from sequencing data with interpretable machine learning. *Nature Biotechnology 2022 40:10* **40**: 1520–1527. https://www.nature.com/articles/s41587-022-01307-0 (Accessed November 8, 2024).

Sandmann T, Girardot C, Brehme M, Tongprasit W, Stolc V, Furlong EEM. 2007. A core transcriptional network for early mesoderm development in Drosophila melanogaster. *Genes Dev* **21**: 436–449. http://genesdev.cshlp.org/content/21/4/436.full (Accessed December 4, 2023).

Sandmann T, Jensen LJ, Jakobsen JS, Karzynski MM, Eichenlaub MP, Bork P, Furlong EEM. 2006. A Temporal Map of Transcription Factor Activity: Mef2 Directly Regulates Target Genes at All Stages of Muscle Development. *Dev Cell* **10**: 797–807.

Starz-Gaiano M, Cho NK, Forbes A, Lehmann R. 2001. Spatially restricted activity of a Drosophila lipid phosphatase guides migrating germ cells. *Development* **128**: 983–991.

Waszak SM, Delaneau O, Gschwind AR, Kilpinen H, Raghav SK, Witwicki RM, Orioli A, Wiederkehr M, Panousis NI, Yurovsky A, et al. 2015. Population Variation and Genetic Control of Modular Chromatin Architecture in Humans. *Cell* **162**: 1039–1050. https://pubmed.ncbi.nlm.nih.gov/26300124/ (Accessed January 24, 2025).

Weirauch MT, Yang A, Albu M, Cote A, Montenegro-Montero A, Drewe P, Najafabadi HS, Lambert SA, Mann I, Cook K, et al. 2015. Determination and Inference of Eukaryotic Transcription Factor Sequence Specificity. *Cell* **158**: 1431–1443.

Zhang Y, Liu T, Meyer CA, Eeckhoute J, Johnson DS, Bernstein BE, Nussbaum C, Myers RM, Brown M, Li W, et al. 2008. Model-based Analysis of ChIP-Seq (MACS). *Genome Biol* **9**: R137.

Zinzen RP, Girardot C, Gagneur J, Braun M, Furlong EEM. 2009. Combinatorial binding predicts spatio-temporal cis-regulatory activity. *Nature* **462**: 65–72.
